# Electrochemical Characterization of Site‐Specifically Metal‐Modified DNA Films on Gold Electrode Surfaces

**DOI:** 10.1002/cplu.202500494

**Published:** 2025-10-09

**Authors:** Nils Flothkötter, Nils Lefringhausen, Daniela Escher, Jens Müller, Heinz‐Bernhard Kraatz

**Affiliations:** ^1^ Institut für Anorganische und Analytische Chemie Universität Münster Corrensstraße 28/30 48149 Münster Germany; ^2^ Department of Physical and Environmental Sciences University of Toronto Scarborough 1265 Military Trail Toronto, Ontario M1C 1A4 Canada; ^3^ Present address: MEET Batterieforschungszentrum Universität Münster Corrensstr. 46 48149 Münster Germany

**Keywords:** charge transfer, copper, deoxyribonucleic acid, metal‐mediated base pairs, surface chemistry

## Abstract

The electrochemical characterization of DNA films with different base mismatches or with Cu^II^‐ or Ag^I^‐mediated pairs was carried out to assess possible immobilization and interaction effects. Toward this end, 3‐hydroxy‐2‐methylpyridin‐4(1*H*)‐one (**H**), imidazole‐4‐carboxylate (**K**), purine‐6‐carboxylate (**P**), and 7‐deaza‐6‐pyrazolylpurine (**D**) were used as artificial metal‐binding nucleobases. Cyclic voltammetry and square‐wave voltammetry confirmed the immobilization of suitably modified oligonucleotides on Au electrodes. The incorporation of the metal ions into the base mismatches to form metal‐mediated base pairs showed a negligible effect on the peak potentials. Ambiguous electrochemical impedance spectroscopy results were obtained for DNA with metal‐mediated base pairs, as some duplexes showed no effect of metal ion addition, while others showed variable charge transfer resistance (*R*_CT_) with no discernible pattern. Notably, the formation of Ag^I^‐mediated base pairs induced larger relative changes in *R*_CT_ compared to Cu^II^‐mediated base pairs. Amongst the latter, only strands containing the artificial nucleobase **H** showed statistically relevant sequence‐ and distance‐dependent charge transfer changes upon metalation. The data indicate that neither nucleobase charge nor nucleobase size directly correlates with the charge transfer resistance, but suggest that changes in DNA film stiffness and hence permeability outweigh other effects.

## Graphical abstract

DNA films containing different Cu^II^‐ or Ag^I^‐mediated base pairs are investigated with respect to their electrochemical properties and how these depend on the size and charge of the artificial metal‐binding nucleobase as well as the location of the metal‐mediated base pair within the duplex.

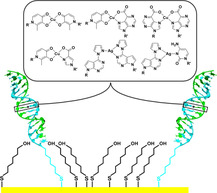

## 1 Introduction

The continuing trend toward miniaturization of electronic devices is leading to a reduction in the size of conductors. As a result, there is a growing interest in exploring wires at the molecular level. Deoxyribonucleic acid (DNA) could be a promising candidate due to its wire‐like structure.^[^ ^1^ ^]^ However, despite its pre‐formed shape, DNA has a relatively low electron transfer rate that decreases by a factor of ca. 10^3^ to 10^4^ every 10 Å, which is significantly slower than, e.g., that of a copper wire.^[^ ^2^ ^]^ In fact, unmodified DNA itself acts as an insulator.^[^ ^3^ ^]^ Nevertheless, charge transfer processes along a DNA duplex are proposed to be involved in biological processes.^[^ ^4^ ^]^ As a result, DNA charge transport remains a topical area of research.^[^ ^5^, ^6^, ^7^, ^8^ ^–^ ^9^ ^]^ The site‐specific introduction of transition metal ions provides an opportunity to modulate the charge transfer ability of DNA, as demonstrated in surface‐deposited individual DNA duplexes bearing site‐specifically bound Ag^I^ or Cu^II^ ions,^[^ ^10^, ^11^ ^–^ ^12^ ^]^ or in solution studies.^[^ ^13^ ^]^ In addition, the interstrand charge transfer in duplexes across a metal cross‐link was evaluated.^[^ ^14^ ^]^

In principle, various strategies are feasible for combining metal ions with nucleic acids. Because of their negatively charged backbone, nucleic acids can adopt their secondary structure only in the presence of suitable cations, typically Na^I^, K^I^, Mg^II^, and organic cations.^[^ ^15^ ^]^ Of course, other cations can also interact with DNA, either nonspecifically or via specific nucleosides designed to bind metal ions.^[^ ^16^ ^]^ Even lanthanoid ions were investigated with respect to their interaction with DNA.^[^ ^17^, ^18^, ^19^, ^20^, ^21^ ^–^ ^22^ ^]^ When appropriately designed, the resulting nucleic acid–metal ion conjugates can be applied as electrochemical sensors for various analytes.^[^ ^23^, ^24^ ^–^ ^25^ ^]^

**Figure 1:**
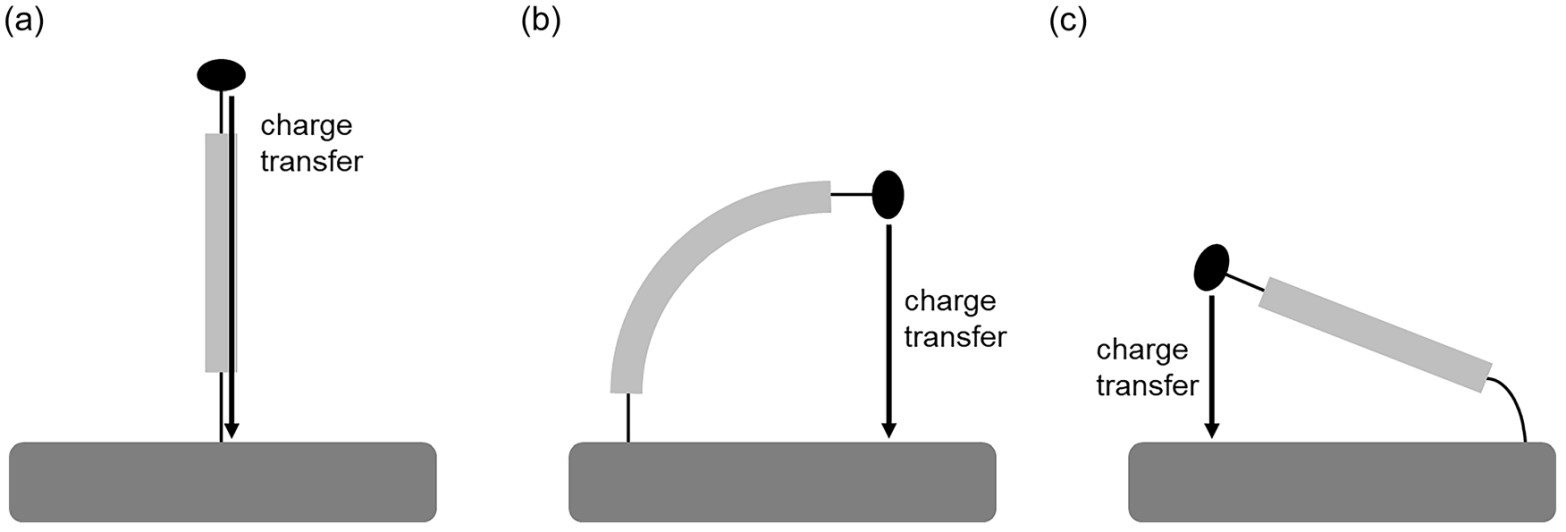
Charge transfer mechanisms of DNA immobilized on Au surfaces via a) direct transfer through the double strand or after bending of b) the dsDNA or c) the linker.

While it is important to study charge transfer across individual double‐stranded DNA (dsDNA) molecules, it is equally important to understand charge transfer in DNA films. The presence of metal ions within the DNA helix significantly affects the charge transfer properties.^[^ ^26^ ^–^ ^28^ ^]^ The formation of a DNA film involves the immobilization of DNA on a substrate surface, which can be achieved by either covalent or electrostatic attachment.^[^ ^29^ ^,^ ^30^ ^]^ Covalent attachment requires a linker moiety at one end of the DNA strands, resulting in the formation of a self‐assembled monolayer once immobilized on the substrate.^[^ ^31^ ^,^ ^32^ ^]^ In many studies, a gold electrode surface is chosen for its ease of fabrication, wide potential window, and reduced chemisorption of DNA.^[^ ^33^ ^]^ The use of a disulfide‐C_6_ linker ensures a straightforward immobilization process via the formation of a stable Au=S bond. This attachment method provides the basis for many applications that rely on charge transfer through these films.^[^ ^34^, ^35^, ^38^ ^–^ ^39^ ^]^ In general, the orientation of immobilized DNA relative to the surface is flexible. Charge transfer can occur directly through the double strand or by bending the strand or linker, when the transfer occurs through the supporting solution (**Figure** **1**).^[^ ^40^ ^]^

The length of the linker and of the duplex plays a key role. Longer duplexes facilitate double‐strand bending and favor bending or folding of the linker. In order to avoid such bending, it is crucial to ensure surface coverage, as the DNA strands need space to bend. Hence, blocking positions where the strands could fold or interact with the Au surface increases the accuracy of an assay. To achieve full surface coverage, 6‐mercapto‐1‐hexanol (MCH) is used to block any remaining positions. Its reduced length compared to the attached DNA potentially favors DNA strand folding over linker bending.^[^ ^27^ ^]^

**Scheme 1:**
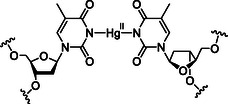
**T**–Hg^II^–**T** as an example for a metal‐mediated base pair (**T **= thymine).

Previous studies on dsDNA films with designated metal‐binding sites had focused primarily on Ag^I^ and Hg^II^. These metal ions had been introduced site‐specifically via the formation of so‐called metal‐mediated base pairs, in which nucleobases are replaced by ligands (**Scheme** **1**).^[^ ^41^ ^]^ Ag^I^‐ and Hg^II^‐mediated base pairing led to changes in surface permittivity, charge transfer resistance, and rate.^[^ ^27^ ^,^ ^36^ ^]^ These effects were reversible upon removal of the incorporated ions. Taking into consideration only dsDNA with metal‐mediated base pairs involving artificial nucleobases, merely films of duplexes with imidazole–Ag^I^–imidazole base pairs were investigated.^[^ ^27^ ^]^ That study showed an increase in charge transfer resistance upon the introduction of the Ag^I^ ions into the imidazole:imidazole mispair, which was explained by the small size of the artificial nucleobases and the concomitant flexibility of the duplex in the absence of stabilizing Ag^I^ ions, so that the incorporation of Ag^I^ ions into the duplex led to its stiffening. Hence, it is important to investigate metal‐mediated base pairs with larger artificial nucleobases.

The present study examines DNA films on gold electrode surfaces, each containing different metal‐binding sites at discrete positions within the dsDNA sequence. It specifically investigates the effects of Cu^II^ binding on duplexes carrying negatively charged or neutral artificial nucleosides in hetero and homo base pairs, respectively. Selected Ag^I^‐mediated base pairs involving the artificial nucleobase 7‐deaza‐6‐pyrazolylpurine (**D**) were also included in the study to allow a comparison among metal‐mediated base pairs of similar sized nucleobase surrogates, albeit with differently charged metal ions.

## 2 Results and Discussion

### 2.1 DNA Duplexes under Investigation

**Scheme 2:**
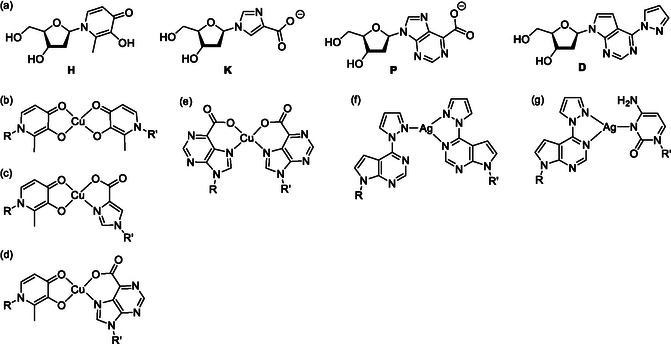
a) Artificial nucleosides as well as b) **H**–Cu^II^–**H**,^[^ ^42^ ^]^ c) **H**–Cu^II^–**K**,^[^ ^43^ ^]^ d) **H**–Cu^II^–**P**, e) **P**–Cu^II^–**P**,^[^ ^45^ ^]^ f) **D**–Ag^I^–**D**,^[^ ^49^ ^]^ and g) **D**–Ag^I^–C base pairs^[^ ^48^ ^]^ investigated in this study.

In this study, a variety of closely related DNA duplexes containing one mispair of one or two artificial nucleobases was investigated. **Table** **1** lists these duplexes, while the artificial nucleoside analogs and the designated metal‐mediated base pairs are displayed in **Scheme** **2**. Nucleobase **H** (3‐hydroxy‐2‐methylpyridin‐4(1*H*)‐one) is well‐known for its ability to form Cu^II^‐mediated base pairs,^[^ ^42^ ^–^ ^44^ ^]^ as are **P** (purine‐6‐carboxylate)^[^ ^45^ ^]^ and **K** (imidazole‐4‐carboxylate).^[^ ^43^ ^,^ ^46^ ^,^ ^47^ ^]^ The formation of an **H**–Cu^II^–**K** pair is known to confer an extraordinary thermal stability to the duplex.^[^ ^43^ ^]^ It is important to note that **H** is a neutral nucleobase, whereas **K** and **P** are negatively charged. All Cu^II^‐mediated base pairs formed from these nucleobases are neutral, as **H** is deprotonated upon metal‐ion binding. Hence, by combining these nucleobases, three possibilities can be realized, because the final neutral Cu^II^‐mediated base pair can be formed from either a neutral **H**:**H** pair, an **H**:**K** or **H**:**P** pair with one negative charge, or from a **K**:**K** or **P**:**P** pair with two negative charges. These three nucleobases are therefore ideally suited to investigate a possible influence of the (change of) charge of a base pair on the charge–transfer ability of a DNA duplex. Moreover, Ag^I^‐mediated base pairs of 7‐deaza‐6‐pyrazolylpurine,^[^ ^48^ ^,^ ^49^ ^]^ which has a size very similar to that of **P** but is neutral, were included in the study to allow a broader comparison.

**Table 1:** DNA duplexes under investigation.

Duplex	Sequence
**XY**_**C**_ a)	R‐5’‐d(TTT G**X**T TGT TTG TTT GTT TTT TTT TT)‐3’ 3’‐d(AAA C**Y**A ACA AAC AAA CAA AAA AAA AA)‐5’
**XY**_**M**_	R‐5’‐d(TTT GTT TGT TTG T**X**T GTT TTT TTT TT)‐3’ 3’‐d(AAA CAA ACA AAC A**Y**A CAA AAA AAA AA)‐5’
**XY**_**F**_	R‐5’‐d(TTT GTT TGT TTG TTT GTT TTT **X**TT TT)‐3’ 3’‐d(AAA CAA ACA AAC AAA CAA AAA **Y**AA AA)‐5’

a) **X** and **Y** denote the nucleobases **H**, **K**, **P, D**, and C. The subscripts C, M, and F refer to the location of the artificial mispair with respect to the gold surface (close, medium, far). R  = –(CH_2_)_6_S_2_(CH_2_)_6_OH. See Table S1 (Supporting Information) for a detailed list of all oligonucleotides.

**Figure 2:**
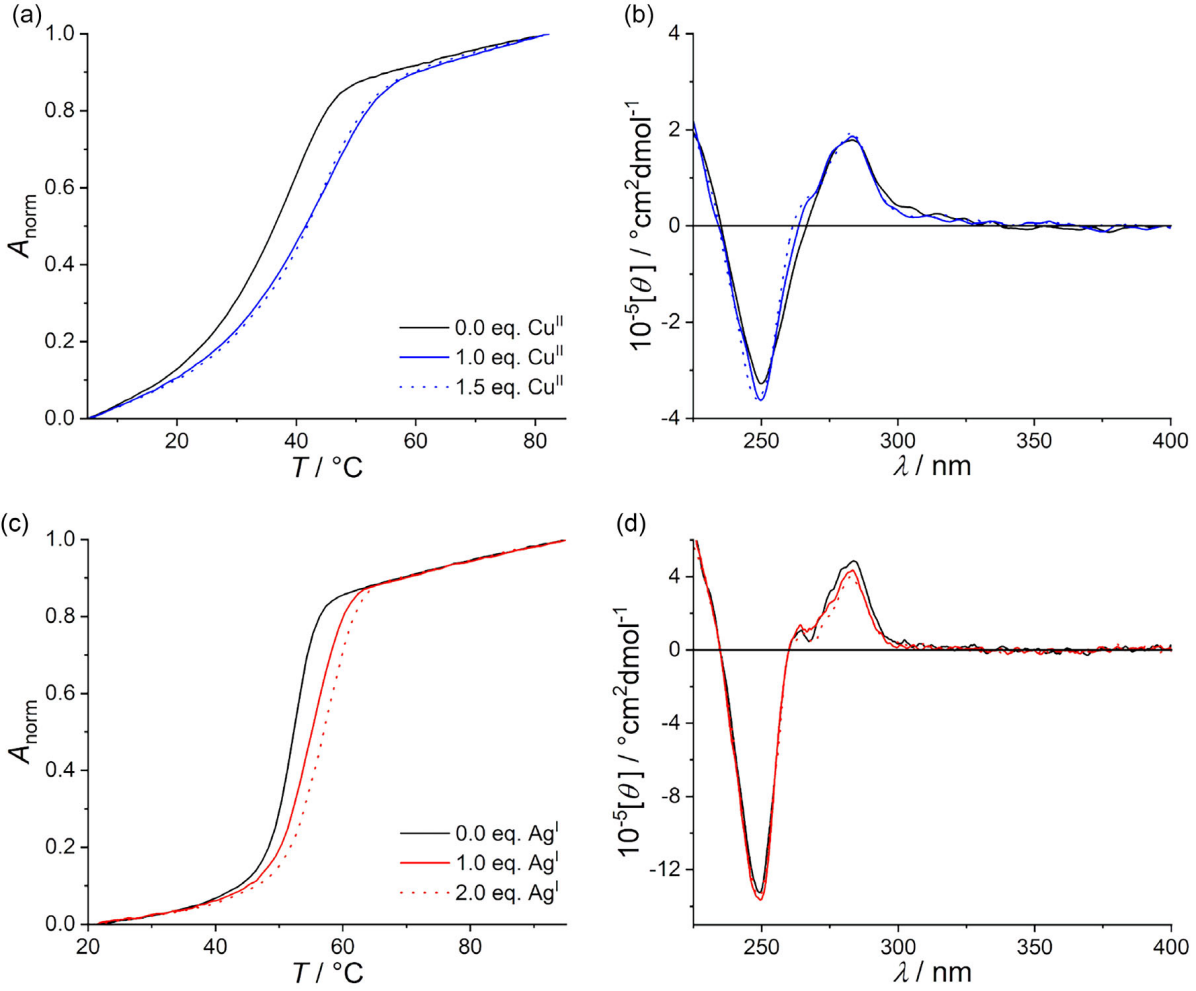
a) Melting curves and b) CD spectra of duplex **PP**_**C**_ in the presence of increasing amounts of Cu^II^. c) Melting curves and d) CD spectra of duplex **CD**_**C**_ in the presence of increasing amounts of Ag^I^. Experimental conditions: 1 μM dsDNA, 5 mM MOPS (pH 6.8), 150 mM NaClO_4_, 1.0 eq. Cu^II^ ≙ 1 μM Cu(NO_3_)_2_, 1.0 eq. Ag^I^ ≙ 1 μM AgNO_3_.

Melting curves and CD spectra were exemplarily recorded in the presence of increasing amounts of Cu^II^ for duplexes **PP**_**C**_, **PP**_**M**_, and **PP**_**F**_ to confirm the anticipated Cu^II^‐mediated base pair formation. **Figure** **2** shows the data obtained for **PP**_**C**_; the other data and the melting temperatures can be found in the Supporting Information (Figure S1, Table S2). As can be discerned from the melting curves, the melting temperature *T*_m_ increases upon the addition of one equivalent of Cu^II^ but does not change significantly in the presence of excess Cu^II^. Such a behavior is indicative of the formation of a Cu^II^‐mediated base pair,^[^ ^50^ ^]^ as it shows the presence of a high‐affinity binding site that binds exactly one Cu^II^ ion. The largest increase in *T*_m_ is observed for **PP**_**M**_ with the central metal‐mediated base pair (Table S2, Supporting Information). The CD spectrum of the duplex resembles that of a typical B‐DNA^[^ ^51^ ^]^ and does not change significantly in the presence of Cu^II^. Hence, the formation of the **P**–Cu^II^–**P** pair does not influence the duplex structure. To further validate that the previously established C–Ag^I^–**D** base pair^[^ ^48^ ^]^ also forms in the oligonucleotide sequence context under investigation here, Figure 2 also shows the melting curves and CD spectra of duplex **CD**_**C**_ in the absence and presence of Ag^I^. The corresponding data for the other Ag^I^‐binding duplexes are shown in the Supporting Information (Figures S2,S3). They indicate that the C–Ag^I^–**D** pair is more stabilizing than the **D**–Ag^I^–**D** pair (Table S2, Supporting Information).

### 2.2 Voltammetry Characterization

**Figure 3:**
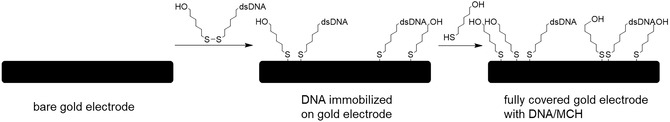
Process of covering a gold electrode surface with a DNA/MCH film using terminally disulfide‐modified DNA and 6‐mercapto‐1‐hexanol.

Cyclic voltammetry (CV) studies were performed to assess DNA binding to gold electrodes and the capping of any remaining potential binding sites with 6‐mercaptohexanol (MCH) (**Figure** **3**). The results are in agreement with expectations, as DNA adsorption blocks the electrode surface, leading to reduced electron flow and reduced accessibility of the Fe^III^/Fe^II^ redox indicator present in solution. This decrease in current confirms a successful duplex adsorption and is accompanied by significant peak broadening and increased peak‐to‐peak separation, indicating an increased potential for charge transfer.

**Figure 4:**
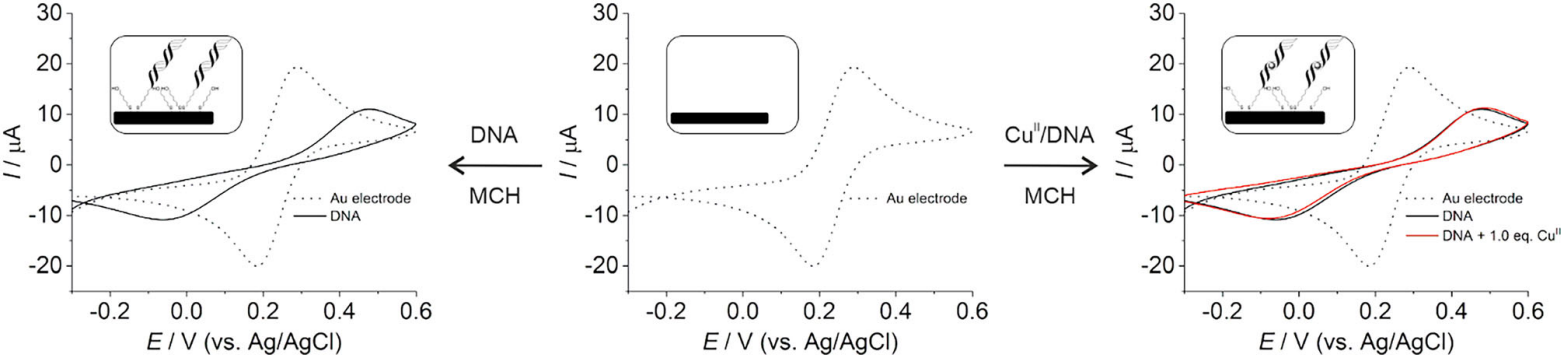
Changes in the cyclic voltammograms of the gold electrode upon deposition of a film of duplex **HH**_**M**_ (left) or of Cu^II^‐containing duplex **HH**_**M**_ (right). The insets depict a schematic view of the surface. Experimental conditions: 2 mM K_4_[Fe(CN)_6_], 2 mM K_3_[Fe(CN)_6_], 5 mM MOPS (pH 6.8), 150 mM NaClO_4_.

Subsequent backfilling of the gold surface with MCH induces a further reduction in current as observed in both CV and square‐wave voltammetry (SWV) experiments, as shown exemplarily for duplex **HH**_**M**_ in **Figure** **4** (data for the other duplexes can be found in the Supporting Information, Figure S4–S10). This reduction in current suggests a reduced accessibility of the Fe^III^/Fe^II^ complexes to the gold surface after DNA immobilization. In addition, the broadening of the peaks and the shift of the maximum further support the notion of an increased potential for charge transfer processes. The presence of Cu^II^ in the DNA has only minimal effects on the voltammograms, in both the CV and SWV experiments.

**Figure 5:**
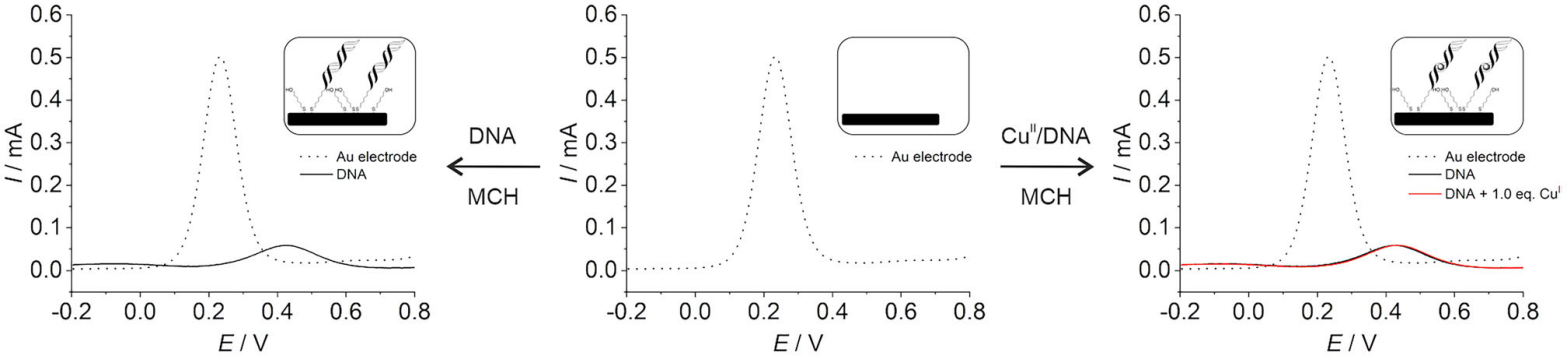
Changes in the square‐wave voltammograms of the gold surface upon deposition of a film of duplex **HH**_**M**_ (left) or of Cu^II^‐containing duplex **HH**_**M**_ (right). The insets depict a schematic view of the surface. Experimental conditions: 2 mM K_4_[Fe(CN)_6_], 2 mM K_3_[Fe(CN)_6_], 5 mM MOPS (pH 6.8), 150 mM NaClO_4_.

The SWV data confirm the observations from the CV experiments, showing a pronounced reduction in current upon DNA/MCH film formation (exemplarily shown for duplex **HH**_**M**_ in **Figure** **5**; data for the other duplexes can be found in the Supporting Information, Figure S11–S17). This reduction is attributed to the reduced availability of Fe^III^/Fe^II^ complexes for redox reactions at the gold surface. In addition, the broadened peaks and the shifted maximum are consistent with an increased potential for charge transfer processes. Again, when a Cu^II^‐mediated base pair is present in the DNA duplex, negligible effects on the voltammograms are observed compared to the Cu^II^‐free duplex, suggesting minimal effects on the electrochemical behavior.

### 2.3 Electrochemical Impedance Spectroscopy (EIS)

**Figure 6:**
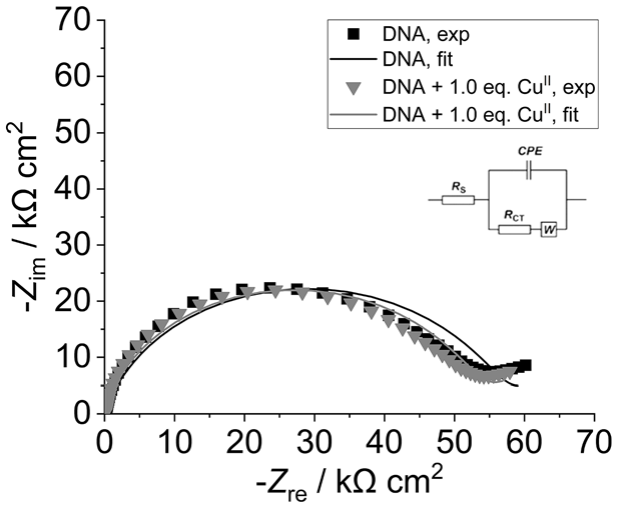
Typical Nyquist plots of a DNA film composed of duplex **HH**_**M**_ in the absence and presence of 1.0 eq. of Cu^II^, based on EIS data. Inserted: Equivalent circuit used to fit the EIS data. Experimental conditions: 2 mM K_4_[Fe(CN)_6_], 2 mM K_3_[Fe(CN)_6_], 5 mM MOPS (pH 6.8), 150 mM NaClO_4_.

To compare the electrochemical properties of the DNA films investigated in this study with previously published results,^[^ ^27^ ^]^ we assessed their charge transfer resistance (*R*_CT_) using EIS. The equivalent circuit used to fit the experimental data is shown in **Figure** **6**. The selection of a suitable equivalent circuit was complicated by irregularities of the semicircle of the experimental data, possibly due to nonuniform surface coating or porosity. We selected an equivalent circuit for its chemical clarity and to ensure consistency with previously published data. In addition, this circuit was found to be the most appropriate one when compared to other models for the same duplexes on Au electrodes (Figure S18, Supporting Information). In this circuit, *R*_s_ represents the solution resistance, while *R*_CT_ represents the charge transfer resistance of the double layer, specifically the DNA/MCH film. The introduction of the constant phase element (*CPE*) takes into consideration the nonuniformity of the DNA/MCH film. In addition, the Warburg impedance accounts for diffusion to and from the electrode surface.

**Figure 7:**
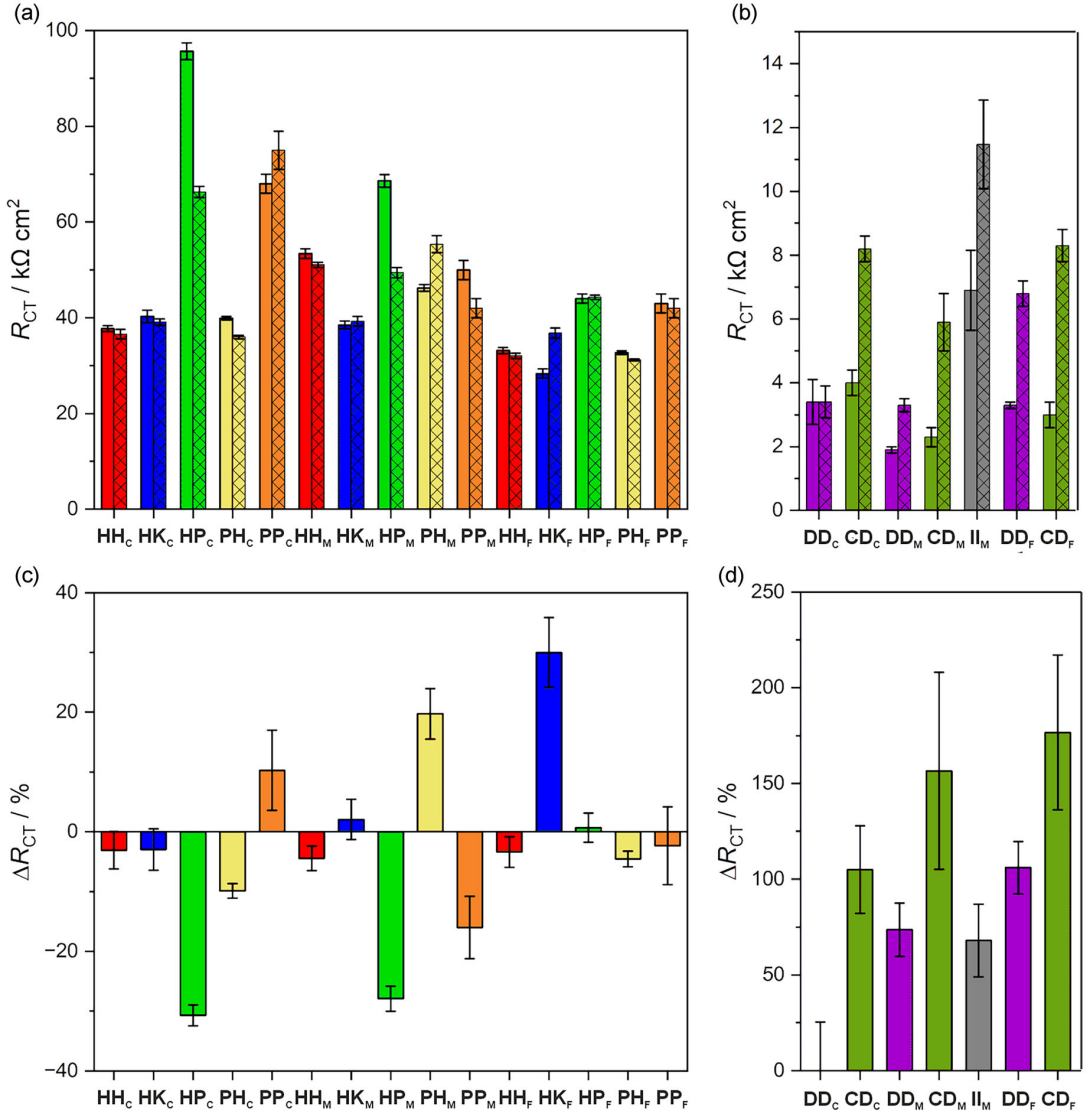
Charge transfer resistance *R*_CT_ (top) of the DNA duplexes in the absence and the presence of one equivalent of Cu^II^ (left) and Ag^I^ (right), and relative changes Δ*R*_CT_ upon the addition of one equivalent of metal ion (bottom). Data for duplex **II**_**M**_ (with one central imidazole–Ag^I^–imidazole base pair) were reported previously.^[^ ^27^ ^]^ Data represent an average of at least three individual measurements.

In the following, the Nyquist plot of **HH**_**M**_ is presented as a representative example (Figure 6). The corresponding plots of the other duplexes are very similar and are compiled in the Supporting Information (Figure S19–S25). The *R*_CT_ values are summarized in Table S3 (Supporting Information). For a better comparison of the different duplexes, a diagrammed summary of the relative change of *R*_CT_ upon the addition of one equivalent of metal ion, i.e., Δ*R*_CT_, is given in **Figure** **7**.

In the absence of Cu^II^ ions, the largest charge transfer resistance is recorded for **HP**_**C**_, amounting to 96(5) kΩ cm^2^. In this duplex, an **H**:**P** mispair is in close proximity to the electrode surface. For **HP**_**C**_, **HP**_**M**_, **HP**_**F**_, **PP**_**C**_, **PP**_**M**_, and **PP**_**F**_, a linear correlation of *R*_CT_ with the distance of the metal‐mediated base pair from the electrode surface is observed, with *R*_CT_ decreasing with increasing distance (Figure 7a, green and orange). Conversely, for **HH**_**C**_, **HH**_**M**_, **HH**_**F**_, **PH**_**C**_, **PH**_**M**_, and **PH**_**F**_, *R*_CT_ adopts its largest value when the artificial base pair is located in the center of the duplex (Figure 7a, red and yellow). For duplexes with an **H**:**K** mispair, hardly any position‐dependence of *R*_CT_ is observed: **HK**_**C**_, **HK**_**M**_, and **HK**_**F**_ show similar *R*_CT_ values regardless of the position of the artificial pair within the duplex (Figure 7a, blue). It hence appears that the identity of the artificial nucleobase in the oligonucleotide not attached to the gold surface determines the trend in *R*_CT_ (**P**: decrease, **H**: peaking at center; **K**: no influence) rather than the identity of the base pair. This is particularly obvious when comparing the duplexes containing **H**:**P** and **P**:**H** mispairs, respectively. The different trends cannot be attributed to the charge of the base pair, as both **P** and **K** are negatively charged, whereas **H** is neutral. It should be noted, though, that **K** is sterically less demanding than **P**, so ligand size may play a role. It is worth noting that for all duplexes studied, *R*_CT_ is the smallest when the artificial base pair is incorporated farthest away from the gold electrode (except for the **D**‐containing duplexes, where this trend is less pronounced; Figure 7a,b). It is likely that in this position the mispair decreases the structural rigidity of the duplex, leading to a reduced film thickness and concomitantly to a smaller *R*_CT_.^[^ ^27^ ^]^ The absolute charge transfer resistance of the DNA films formed from the Ag^I^‐binding duplexes in the absence of Ag^I^ (Figure 7b) is smaller than that of the Cu^II^‐binding duplexes (Figure 7a), likely a result of the use of a different electrode.

With respect to any trends upon the incorporation of metal ions, *R*_CT_ had previously been observed to increase upon the incorporation of Ag^I^ into dsDNA.^[^ ^27^ ^,^ ^36^ ^]^ This is confirmed for all Ag^I^‐containing duplexes investigated here (Figure 7b), with the exception of duplex **DD**_**C**_, for which no relevant change was detected. Interestingly, no increase in *T*_m_ was observed for this duplex upon the addition of Ag^I^ either (Figure S2, Supporting Information). No clear trend can be discerned for the Cu^II^‐binding duplexes (Figure 7c). Regarding the absolute values of *R*_CT_, the overall trend observed for the Cu^II^‐free films remains unaltered, namely that the identity of the artificial nucleobase in the oligonucleotide not attached to the gold surface determines the trend in *R*_CT_ (**P**: decrease, **H**: peaking at center; **K**: no influence; Figure 7a).

As the absolute values of *R*_CT_ appear to be influenced by the electrode used in the investigations, the following discussion will focus on relative changes in *R*_CT_ upon metalation. For eight of the sixteen Cu^II^‐binding duplexes investigated (**HH**_**C**_, **HH**_**M**_, **HH**_**F**_, **HK**_**C**_, **HK**_**M**_, **HP**_**F**_, **PH**_**F**_, **PP**_**C**_), *R*_CT_ does not change significantly upon Cu^II^ binding. As the mispairs in these duplexes are neutral (**H**:**H**), anionic (**H**:**K**, **H**:**P**, **P**:**H**), or even dianionic (**P**:**P**), whereas the Cu^II^‐mediated base pairs are neutral in all cases, charge appears not to be a relevant factor influencing Δ*R*_CT_. Also taking into consideration the CV and SWV data, it can be concluded that the incorporation of Cu^II^ into the dsDNA does not significantly affect the charge transfer properties of these eight duplexes.

In contrast, duplexes **PH**_**M**_ and **HK**_**F**_ with a **P**–Cu^II^–**H** or an **H**–Cu^II^–**K** pair show an increase in charge transfer resistance by 20% ± 4% and 30% ± 6%, respectively, suggesting a significant effect of Cu^II^ binding. A less significant increase of 10% ± 7% is observed for **PP**_**C**_. It is interesting to note that the largest increase in *R*_CT_ is observed upon formation of the extraordinarily stable **H**–Cu^II^–**K** pair^[^ ^43^ ^]^ at a location farthest away from the gold surface. This suggests that the formation of this Cu^II^‐mediated base pair stiffens the duplex, thereby increasing DNA film thickness and making the film less permeable for the redox probe, finally leading to an increase in *R*_CT_.^[^ ^27^ ^]^

A decreasing *R*_CT_ is observed for **HP**_**C**_ (–31% ± 2%) and **HP**_**M**_ (–28% ± 2%), and to a lesser extent for **PP**_**M**_ (–16% ± 5%). In the case of the former two duplexes, the decrease correlates well with the largest *R*_CT_ prior to metalation. Here, a change of duplex stiffness upon metalation is less likely. Instead, it might be speculated that the change from an anionic mispair to a neutral metal‐mediated base pair also involves a change in surface charge of the DNA monolayers and thereby influences *R*_CT_.^[^ ^52^ ^]^ However, such a rationalization does not explain why this trend is observed for selected anionic mispairs only.

A comparison with Δ*R*_CT_ of the Ag^I^‐containing duplexes may help evaluate whether ligand size is a relevant factor in determining Δ*R*_CT_, because the ligand 7‐deaza‐6‐pyrazolylpurine (**D**) used in the Ag^I^‐mediated base pairs has approximately the same size as purine‐6‐carboxylate (**P**) used in the Cu^II^‐mediated base pairs, but is neutral. As can be seen from Figure 7d, the incorporation of Ag^I^ into the duplexes leads to an increase in *R*_CT_ (except for **DD**_**C**_, *vide supra*), which roughly correlates with an increase in *T*_m_ (Table S2, Supporting Information). The relative increase is much larger than for the Cu^II^‐containing duplexes (up to 180% ± 40%). Nevertheless, it is in the same order of magnitude as observed previously for DNA films with an imidazole–Ag^I^–imidazole base pair (**II**_**M**_ in Figure 7d).^[^ ^27^ ^]^ Still, this latter comparison indicates that there is no obvious correlation between nucleobase size and Δ*R*_CT_: The change in *R*_CT_ upon incorporation of a silver ion into **DD**_**M**_ (70% ± 10%) is essentially identical to the one upon incorporation of a silver ion into the much smaller **II**_**M**_ (70% ± 20%), with both mispairs being neutral. Hence, even though a direct comparison between the Cu^II^‐ and the Ag^I^‐containing duplexes reported here appears to be less constructive, nucleobase size can be considered a less relevant factor influencing the charge transfer resistance.

In general, the trends observed here are similar to the ones reported when studying the influence of mismatched canonical nucleobases on *R*_CT_. Those studies demonstrated that *R*_CT_, as measured by EIS or scanning electrochemical microscopy (SECM), varies significantly between a fully matched duplex supported on an electrode surface and duplexes containing one or more mispairs.^[^ ^53^ ^]^ Mismatch films show lower or altered charge transfer resistances than perfectly matched DNA films. The mismatched base pairs disrupt helix structure, allowing more access of the redox mediator to the electrode surface.^[^ ^52^ ^]^ The position of a given mismatch within the oligonucleotide duplex influences the magnitude of the changes in *R*_CT_. Base mismatches proximal to the electrode surface or in the middle behave differently from those at the distal ends of duplexes. This has been rationalized by a significant variation in the access of redox mediators to the electrode, changes in the flexibility of the duplex DNA, and the oligonucleotide packing density, all of which depend on the mismatch position.^[^ ^37^ ^,^ ^54^ ^]^ Later studies demonstrated the ability to detect a single nucleotide mismatch distal from the electrode surface,^[^ ^55^ ^]^ also expanded to non‐Watson Crick base pairing as in quadruplex DNA structures.^[^ ^56^ ^]^ For the canonical nucleobases, the value of Δ*R*_CT_ depends both on which bases are mismatched and where in the duplex they are located. For example, a purine‐pyrimidine mismatch in the middle of a duplex results in a larger change in *R*_CT_ compared to other positions.^[^ ^39^ ^,^ ^57^ ^]^ Detailed SECM studies have demonstrated the use of this approach for the detection of single‐nucleotide mismatches and position variations in larger DNA fragments, aimed at developing a tool for the identification of species using genomic and mitochondrial oligonucleotide fragments.^[^ ^58^, ^59^ ^–^ ^60^ ^]^

## 3 Conclusion

The electrochemical characterization of a series of DNA films containing either one mispair, one Cu^II^‐mediated base pair, or one Ag^I^‐mediated base pair shows ambiguous results with respect to the influence of the metal ion on the charge transfer resistance *R*_CT_ of the film. In particular, the overall charge of the mispair/ metal‐mediated base pair appears to play only a minor role in determining *R*_CT_, given the different results obtained for duplexes containing **K** and **P**. Likewise, the size of the mispair has a negligible influence on Δ*R*_CT_, as shown by a comparison of Ag^I^‐mediated base pairs. Unexpectedly, the identity of a mispair (or Cu^II^‐mediated base pair) has less of an influence on *R*_CT_ than the identity of the artificial nucleobase located in the oligonucleotide strand which is not connected to the gold surface: for **P**, *R*_CT_ decreases with increasing distance of the mispair from the gold surface, while is peaks at medium distance for **H**, with **K** showing no significant distance‐dependence at all. Nevertheless, in almost all cases, *R*_CT_ is smallest when the pair is incorporated far away from the electrode, suggesting that the charge transfer resistance of the DNA films (with and without metal ions) mainly correlates with the film stiffness.

## 4 Experimental Section

4.1

4.1.1

Reagents and solvents were used without further purification and purchased from *Acros Organics*, *ABCR*, *Sigma–Aldrich*, *Fisher Scientific*, *Merck*, *VWR*, *Carl Roth GmbH & Co. KG*, *Glen Research*, *TCI*, and *Eurogentec*. Whenever absolute solvents were needed, they were dried and distilled according to standard procedures and stored over molecular sieves. Aqueous solutions were prepared with doubly deionized water (18 MΩ cm^–1^ resistivity). Silica gel used for column chromatography was purchased from *Acros Organics* with a pore size of 60 Å and a particle size of 35–70 μm. Thin‐layer chromatography plates were obtained from *Merck* (DC Silica Gel 60 F254) and were analyzed using UV light (*λ* = 254 nm).

^1^H NMR and ^13^C NMR spectra were recorded at 300 K on *Bruker* Avance(I) 400, Avance(III) 400, Avance NEO 400, and Avance NEO 500 spectrometers. For referencing, tetramethylsilane (TMS) was used for CDCl_3_ solutions. All other samples, dissolved in CD_2_Cl_2_ or DMSO‐*d*_6_, were referenced using the residual solvent signals.^[^ ^61^ ^]^^15^N and ^31^P NMR spectra were recorded at 300 K on *Bruker* Avance(III) 400 or Avance NEO 500 spectrometers and referenced according to IUPAC using ammonia and phosphoric acid (*c* = 85%) as internal standards, respectively. Elemental analyses were carried out on a Vario EL III CHNS analyzer.

Oligonucleotides were synthesized using a K&A Laborgeräte H8 DNA/RNA synthesizer at a 1 μmol scale, following standard protocols for solid‐phase DNA synthesis with the phosphoramidite method. Oligonucleotides purified by gel electrophoresis were synthesized in “DMT‐off” mode, while other oligonucleotides were synthesized in “DMT‐on” mode. A coupling time of 100 s was used for natural nucleosides, and 1000s for artificial nucleosides dissolved in acetonitrile (0.1 M solution). Commercially available reagents, including CPGs and phosphoramidites of natural nucleosides, were purchased from *Alfa Aesar*, *Fluka*, *GlenResearch*, *Carl Roth GmbH & Co. KG*, *SAFC*, *Sigma–Aldrich*, or *VWR.* The deprotection and cleavage from the solid support took place after the synthesis of the oligonucleotides and was conducted under basic conditions. For the artificial nucleosides **K** and **P**, treatment with 0.1 M NaOH for 18 h at 55 °C was chosen, while for **H** and **D**, aqueous ammonia (25% in water) for 12 h at 55 °C was sufficient. Purification of the oligonucleotides was achieved via polyacrylamide gel electrophoresis. Urea (7 M), TBE buffer solution (1 M in water), and 14% to 18% polyacrylamide:bisacrylamide (29:1) were used. As a loading buffer, a mixture of urea (11.8 M), Tris‐HCl (42 mM, pH 7.5), EDTA (0.83 mM in water, pH 8.0), and sucrose (8%) was employed, along with xylene cyanole (0.08%) and bromophenol blue (0.08%) as markers. A power of 35 W per gel was applied. Subsequently, the DNA was eluted from the gel pieces using electrolution (200 V) in TB or TBE buffer. Desalting was performed once or twice using NAP‐10 columns (Sephadex G‐25 DNA grade) from *Fisher Scientific*. Oligonucleotides containing the disulfide modifier were purified using reverse‐phase high‐performance liquid chromatography using an instrument from *Jasco* (PU‐2080 PLUS) and a Platinum C18 EPS (4.6 × 150 mm, 3 μm) column heated to 50 °C with a flow rate of 1 mL min^–1^. The following gradient of buffer A (10 mM triethylammonium acetate in water, pH 7.0) and buffer B (10 mM triethylammonium acetate in acetonitrile/water 80/20, pH 7.0) was used: 0–5 min, 0–3% B; 5–25 min, 3–25% B. A *Thermo Scientific* NanoDrop 2000c spectrophotometer was used to quantify DNA concentrations. To ensure reversibility, the duplexes were annealed by heating the solution to 85 °C and then gradually cooling them to 5 °C at a rate of 1 °C min^–1^ before each experiment. Experimental data were processed using OriginPro (*OriginLab*).

CHI 101 gold disk electrodes were purchased from *CH Instruments* (Austin, TX), with a radius of 1 mm. Prior to any experiments, the gold electrodes underwent polishing using alumina slurries (particle sizes 1, 0.3, and 0.05 μm) for 3 min each. Subsequently, the electrodes were ultrasonicated in doubly deionized water, followed by ethanol, and again in doubly deionized water for 10 min each. Electrochemical cleaning was then performed: first in aqueous KOH solution (0.5 M, 0–1.5 V with a rate of 0.5 V s^–1^), and subsequently in aqueous H_2_SO_4_ solution (0.5 M, 0–1.5 V with a rate of 0.5 V s^–1^), until reproducibility was achieved, but at least for 10 min. After the final cleaning step, the bare gold electrodes were measured under experimental conditions. If no impurities were detected, they were used for further experiments. The incubation was carried out using a DNA stock solution containing 25 μM dsDNA, 150 mM NaClO_4_, and 5 mM MOPS (pH 6.8) for 3 days at 5 °C. Subsequently, the surfaces were carefully washed with buffer solution. For sulfide linker strands, a set of experiments (CV, SWV, and EIS) was conducted to demonstrate the success of the incubation. The incubation for dsDNA containing metal ions was carried out using stock solutions containing 25 μM dsDNA, 25 μM Cu^II^ or Ag^I^ (1.0 eq. with respect to mispairs), 150 mM NaClO_4_, and 5 mM MOPS (pH 6.8) for 3 days at 5 °C. The metal‐mediated base pairs were formed by heating the mixture to 70 °C and controlled cooling with 1 °C min^–1^. Metal‐mediated base pair formation took place before attachment of the metalated dsDNA to the electrode. Care was taken to ensure that the same electrodes were used for measurements in the absence and presence of metal ions. In general, the electrode surfaces were capped with 6‐mercapto‐1‐hexanol (MCH) for 1 hr at 5 °C (10 mM MCH, 150 mM NaClO_4_, 5 mM MOPS (pH 6.8)). After another washing step with buffer solution, CV, square wave voltammetry, and EIS experiments were performed in a Faraday cage using a CHI‐6059E potentiostat from *CH instruments*. The three‐electrode electrochemical cells were self‐fabricated, with the gold electrode as the working electrode, a platinum wire as the counter electrode, and an Ag/AgCl electrode (3 M in KCl) as the reference electrode. Both individual vessels were connected with the appropriate wiring. Prior to each electrochemical experiment, the Au electrodes were cleaned mechanically and electrochemically. All electrochemical experiments were conducted in triplicate to reduce experimental error, to account for potential impurities on the gold electrodes, and to ensure reproducibility. Mean values and standard deviations are provided for all experiments. CV measurements were conducted within a potential range of –0.3 to 0.6 V at a scan rate of 0.1 V s^–1^. Peak potentials were determined by calculating the derivative of the corresponding currents (*dip*(*E*)/*dE* = 0) using OriginPro (*OriginLab*). For SWV experiments, the initial potential was set to –0.2 V, and the final potential to 0.8 V. The increment potential was 4 mV, the pulse amplitude was 25 mV, and the pulse frequency was 15 Hz. EIS studies utilized an open circuit potential as the initial potential for actual measurements. The data were recorded across a frequency range of 100 to 0.1 MHz with an amplitude of 5 mV. Data evaluation was performed using ZSimpWin 2.0 by *AMETEK Scientific* Instruments, and the plots were generated using OriginPro (*OriginLab*). The average charge transfer resistance, along with its standard deviation, was calculated in Microsoft Excel based on at least three individual measurements.

Nucleosides **H**, **D**, and **P** and their respective phosphoramidite derivatives required for automated solid‐phase oligonucleotide synthesis were prepared as described in the literature,^[^ ^42^ ^,^ ^46^ ^]^ whereas the synthesis of nucleoside **K** was performed according to a modified literature procedure (Scheme S1, Supporting Information).^[^ ^45^ ^]^

**3′,5′‐*O*‐(1,1,3,3‐tetraisopropyldisiloxane‐1,3‐diyl)‐2′‐deoxyadenosine (1).** 2′‐Deoxyadenosine (1.00 g, 4.98 mmol, 1.0 eq.), imidazole (0.61 g, 9.0 mmol, 1.8 eq.), and DMAP (0.09 g, 0.7 mmol, 0.1 eq.) were dissolved in dry DMF (20 mL), and 1,3‐dichloro‐1,1,2,2‐tetraisopropyl disiloxane (1.50 mL, 1.48 g, 4.69 mmol, 0.9 eq.) was added slowly to the solution. The mixture was stirred at room temperature overnight and treated with water (50 mL). The crude product was extracted with DCM (3 × 50 mL) and purified by flash chromatography (DCM:MeOH 50:1) and obtained as a white foam (2.16 g, 4.38 mmol, 88%). ^**1**^ **H NMR** (400 MHz, CDCl_3_) *δ* = 8.29 (s, 1H, H2), 8.01 (s, 1H, H8), 6.28 (dd, ^3^ *J*_HH_ = 7.4 Hz, ^3^ *J*_HH_ = 2.6 Hz, 1H, H1′), 6.21 (s, 2H, NH_2_), 4.94 (dt, ^3^ *J*_HH_ = 9.0 Hz, ^3^ *J*_HH_ = 7.4 Hz, 1H, H3’), 4.04 (m, 2H, H5’, H5″), 3.88 (dt, ^3^ *J*_HH_ = 7.8 Hz, ^3^ *J*_HH_ = 4.0 Hz, 1H, H4’), 2.71 (ddd, ^2^ *J*_HH_ = 13.3 Hz, ^3^ *J*_HH_ = 7.5 Hz, ^3^ *J*_HH_ = 2.7 Hz, 1H, H2’ or H2″), 2.62 (ddd, ^2^ *J*_HH_ = 13.3 Hz, ^3^ *J*_HH_ = 9.0 Hz, ^3^ *J*_HH_ = 7.4 Hz, 1H, H2’ or H2″), 1.12–0.99 (m, 28H, ^*i*^Pr) ppm. ^**13**^ **C{H} NMR** (101 MHz, CDCl_3_) *δ* = 155.6 (C6), 152.8 (C2), 149.0 (C4), 138.9 (C8), 120.2 (C5), 85.2 (C4’), 83.2 (C1′), 69.9 (C3’), 61.8 (C5’), 40.1 (C2’), 17.5 (^*i*^Pr‐CH_3_), 17.4 (2 × ^*i*^Pr‐CH_3_), 17.3 (^*i*^Pr‐CH_3_), 17.1 (^*i*^Pr‐CH_3_), 17.0 (2 × ^*i*^Pr‐CH_3_), 16.9 (^*i*^Pr‐CH_3_), 13.4 (^*i*^Pr‐CH), 13.1 (^*i*^Pr‐CH), 12.8 (^*i*^Pr‐CH), 12.5 (^*i*^Pr‐CH) ppm. ^**15**^ **N NMR** (41 MHz, CDCl_3_) *δ* = 236 (N7), 233 (N1), 227 (N3), 177 (N9), 74 (NH_2_) ppm. ^**29**^ **Si NMR** (79 MHz, CDCl_3_) *δ* = –13.2 (Si–O5’), –11.3 (Si–O3’) ppm. **ESI(+)‐MS** [M + H]^+^ calculated: 494.2599 found: 494.2613. **Elemental analysis:** Chemical formula: C_22_H_39_N_5_O_4_Si_2_: C 53.4; H 7.9; N 13.7; calcd. C 53.5; H 8.0; N 14.2.

**6‐Iodo‐3′,5′‐*O*‐(1,1,3,3‐tetraisopropyldisiloxane‐1,3‐diyl)‐*β*‐D‐2′‐deoxyribosylpurine (2).** A solution of the protected nucleoside **1** (1.00 g, 2.03 mmol, 1.0 eq.), iodine (0.23 g, 0.91 mmol, 0.5 eq.), and isoamyl nitrite (0.95 μL, 0.82 g, 0.70 mmol, 0.3 eq.) in THF (30 mL) was heated to 60 °C. After stirring the solution for 2 days, the solvent was removed, and the residue was purified by flash chromatography (CH:EtOAc 4:1) to yield a white–yellow foam (1.01 g, 1.67 mmol, 82%). ^**1**^ **H NMR** (400 MHz, CDCl_3_) *δ* = 8.55 (s, 1H, H2), 8.31 (s, 1H, H8), 6.30 (dd, ^3^ *J*_HH_ = 7.4 Hz, ^3^ *J*_HH_ = 2.5 Hz, 1H, H1′), 4.93 (q, ^3^ *J*_HH_ = 7.8 Hz, 1H, H3’), 4.01 (d, ^3^ *J*_HH_ = 3.9 Hz, 2H, H5’, H5″), 3.88 (dt, ^3^ *J*_HH_ = 7.7 Hz, ^3^ *J*_HH_ = 3.9 Hz, 1H, H4’), 2.75 (ddd, ^2^ *J*_HH_ = 13.5 Hz, ^3^ *J*_HH_ = 7.5 Hz, ^3^ *J*_HH_ = 2.5 Hz, 1H, H2’ or H2″), 2.66 (ddd, ^2^ *J*_HH_ = 13.4 Hz, ^3^ *J*_HH_ = 9.1 Hz, ^3^ *J*_HH_ = 7.5 Hz, 1H, H2’ or H2″), 1.04 (m, 28H, ^*i*^Pr) ppm. ^**13**^ **C{H} NMR** (101 MHz, CDCl_3_) *δ* = 151.7 (C2), 146.8 (C4), 143.0 (C8), 139.4 (C5), 122.2 (C6), 85.3 (C4’), 83.7 (C1′), 69.7 (C3’), 61.5 (C5’), 39.8 (C2’), 17.4 (2 × ^*i*^Pr‐CH_3_), 17.3 (2 × ^*i*^Pr‐CH_3_), 17.1 (^*i*^Pr‐CH_3_), 17.0 (^*i*^Pr‐CH_3_), 16.9 (^*i*^Pr‐CH_3_),16.8 (^*i*^Pr‐CH_3_), 13.3 (^*i*^Pr‐CH), 13.0 (^*i*^Pr‐CH), 12.8 (^*i*^Pr‐CH), 12.5 (^*i*^Pr‐CH) ppm. ^**15**^ **N NMR** (41 MHz, CDCl_3_) *δ* = 297 (N1), 247 (N3), 179 (N9) ppm. ^**29**^ **Si NMR** (400 MHz, CDCl_3_) *δ* = –13.0 (Si–O5’), –11.2 (Si–O3’) ppm. **ESI(+)‐MS** m/z [M+H]^+^ calculated: 605.1471 found: 605.1474. **Elemental analysis**: Chemical formula: C_22_H_37_IN_4_O_4_Si_2_: C 43.9; H 5.9; N 9.2, calcd. C 43.7; H 6.2; N 9.3.

**3′,5′‐*O*‐(1,1,3,3‐tetraisopropyldisiloxane‐1,3‐diyl)‐*β*‐D‐2′‐deoxyribosylpurine‐6‐carboxylic acid methyl ester (3).** 6‐Iodo‐3′,5′‐*O*‐(1,1,3,3‐tetraisopropyldisiloxane‐1,3‐diyl)‐*β*‐D‐2′‐deoxyribosylpurine (1.67 g, 2.76 mmol, 1.0 eq.) and 1,1′‐bis(diphenylphosphino)ferrocene (0.22 g, 0.40 mmol, 0.1 eq.) were dissolved in dry acetonitrile (15 mL), dry MeOH (15 mL) and dry triethylamine (1 mL). The suspension was degassed, then (1,1′‐bis(diphenylphosphino)ferrocene) dichloro palladium(II) (0.12 g, 0.16 mmol, 0.1 eq.) was added. After freezing the reaction mixture, the argon atmosphere was removed in vacuo and replaced by carbon monoxide (1 bar). The tube was sealed, and the mixture was allowed to warm to room temperature and then stirred for 2 days at 65 °C. The suspension was cooled to room temperature afterward, and the carbon monoxide atmosphere was removed by a flow of argon. The released gas was burned. Furthermore, the reaction mixture was purged with argon for at least 2 hr until no carbon monoxide could be detected in the gas flow, and the remaining solvent was removed in vacuo. The crude product was purified by column chromatography (DCM:MeOH 100:1) and subsequently recrystallized in hexane (30 mL) to yield colorless crystals of compound **3** (1.19 g, 2.22 mmol, 80%). ^**1**^ **H NMR** (400 MHz, CDCl_3_) *δ* = 9.07 (s, 1H, H2), 8.47 (s, 1H, H8), 6.40 (dd, ^3^ *J*_HH_ = 7.3 Hz, ^3^ *J*_HH_ = 2.4 Hz, 1H, H1′), 4.96 (m, 1H, H3’), 4.12 (s, 3H, OCH_3_), 4.04 (d, ^3^ *J*_HH_ = 3.9 Hz, 2H, H5’, H5″), 3.91 (dt, ^3^ *J*_HH_ = 7.6 Hz, ^3^ *J*_HH_ = 3.9 Hz, 1H, H4’), 2.84–2.73 (m, 2H, H2’, H2″), 1.16–0.83 (m, 28H, ^*i*^Pr) ppm. ^**13**^ **C{H} NMR** (101 MHz, CDCl_3_) *δ* = 163.9 (C=O), 153.0 (C4), 152.0 (C2), 146.1 (C8), 145.0 (C6), 133.2 (C5), 85.4 (C4’), 83.5 (C1′), 69.7 (C3’), 61.5 (C5’), 53.4 (OCH_3_), 39.9 (C2’), 17.5 (^*i*^Pr‐CH_3_), 17.4 (2 × ^*i*^Pr‐CH_3_), 17.3 (^*i*^Pr‐CH_3_), 17.1 (^*i*^Pr‐CH_3_), 17.0 (^*i*^Pr‐CH_3_), 16.9 (2 × ^*i*^Pr‐CH_3_), 13.4 (^*i*^Pr‐CH), 13.1 (^*i*^Pr‐CH), 12.9 (^*i*^Pr‐CH), 12.5 (^*i*^Pr‐CH) ppm. ^**29**^ **Si NMR** (79 MHz, CDCl_3_) *δ* = –12.9 (Si–O5’), –11.1 (Si–O3’) ppm. **ESI(+)‐MS** m/z [M + H]^+^ calculated: 537.2559 found: 537.2544. **Elemental analysis**: Chemical formula: C_24_H_40_N_4_O_6_Si_2_: C 53.4; H 7.3; N 10.2; calcd. C 53.7; H 7.5; N 10.4.

***β*‐D‐2′‐deoxyribosylpurine‐6‐carboxylic acid methyl ester (4).** Triethylamine trihydrofluoride (1.07 g, 6.64 mmol) was added dropwise at 0 °C to a solution of compound **3** (1.42 g, 2.65 mmol) in dry THF (20 mL). After stirring the solution for 8 hr at room temperature, CaSO_4_ (200 mg) was added, and the solvent was removed in vacuo and the crude product was purified by column chromatography (DCM:MeOH 20:1) to yield 3′,5′‐*O*‐(1,1,3,3‐tetraisopropyldisiloxane‐1,3‐diyl)‐*β*‐D‐2′‐deoxyribosylpurine‐6‐carboxylic acid methyl ester as a white solid (0.76 g, 2.6 mmol, 96%). ^**1**^ **H NMR** (400 MHz, DMSO‐d_6_) *δ* = 9.06 (s, 1H, H2), 8.96 (s, 1H, H8), 6.52 (t, ^3^ *J*_HH_ = 6.6 Hz, 1H, H1′), 5.36 (d, ^3^ *J*_HH_ = 4.3 Hz, 1H, 3′‐OH), 4.96 (t, ^3^ *J*_HH_ = 5.5 Hz, 1H, 5′‐OH), 4.46 (dq, ^3^ *J*_HH_ = 7.5, 3.9 Hz, 1H, H3’), 3.98 (s, 3H, OCH_3_), 3.91 (td, ^3^ *J*_HH_ = 4.4, 3.2 Hz, 1H, H4’), 3.63 (m, 1H, H5’ or H5″), 3.54 (ddd, ^2^ *J*_HH_ = 11.8 Hz, ^3^ *J*_HH_ = 5.6, 4.5 Hz, 1H, H5’ or H5″), 2.79 (dt, ^2^ *J*_HH_ = 13.0 Hz, ^3^ *J*_HH_ = 6.4 Hz, 1H, H2’ or H2″), 2.39 (ddd, ^2^ *J*_HH_ = 13.4 Hz, ^3^ *J*_HH_ = 6.4, 3.9 Hz, 1H, H2’ or H2″) ppm. ^**13**^ **C{H} NMR** (126 MHz, DMSO‐d_6_) *δ* = 163.7 (C=O), 153.1 (C4), 151.4 (C2), 147.2 (C8), 144.7 (C6), 131.9 (C5), 88.0 (C4’), 83.8 (C1′), 70.3 (C3’), 61.3 (C5’), 52.7 (OCH3), 39.3 (C2’) ppm. **ESI(+)‐MS m/z** [M + H]^+^ calculated: 537.2559 found: 537.2544. Elemental analysis: C_12_H_14_N_4_O_5_: C 48.8; H 4.5; N 18.7; calcd. C 49.0; H 4.8; N 19.0.

**5′‐*O*‐(4,4′‐dimethoxytrityl)‐*β*‐D‐2′‐deoxyribosylpurine‐6‐carboxylic acid methyl ester (5).** *β*‐D‐2′‐deoxyribosylpurine‐6‐carboxylic acid methyl ester (0.83 g, 2.8 mmol, 1.0 eq.) was dissolved in dry pyridine (30 mL). After stirring the solution for 10 min, 4,4′‐dimethoxytriphenylmethyl chloride (1.07 g, 3.16 mmol, 1.1 eq.) was added and the mixture was stirred for another 8 hr. The reaction mixture was quenched with MeOH (5 mL), and the solvent was evaporated in vacuo. The residue was purified by column chromatography (DMC:MeOH:NEt_3_ 50:1:1) and subsequent recrystallization from EtOAc yielded compound **5** as a white foam (1.31 g, 2.20 mmol, 78%). ^**1**^ **H NMR** (400 MHz, CD_2_Cl_2_) *δ* = 8.96 (s, 1H, H2), 8.45 (s, 1H, H8), 7.41 (m, 2H, DMT), 7.33–7.27 (m, 4H, DMT), 7.27–7.15 (m, 3H, DMT), 6.80–6.76 (m, 4H, DMT), 6.57 (t, ^3^ *J*_HH_ = 6.5 Hz, 1H, H1′), 4.71 (dt, ^3^ *J*_HH_ = 6.1, 3.7 Hz, 1H, H3’), 4.26 (q, ^3^ *J*_HH_ = 4.4 Hz, 1H, H4’), 4.05 (s, 3H, OCH_3_), 3.75 (s, 6H, 2 × OCH_3_ (DMT)), 3.38 (dt, ^3^ *J*_HH_ = 7.6, 4.0 Hz, 2H, H5’, H5″), 2.85 (dt, ^2^ *J*_HH_ = 13.0 Hz, ^3^ *J*_HH_ = 6.3 Hz, 1H, H2’ or H2″), 2.71–2.63 (m, 1H, H2’ or H2″) ppm. ^**13**^ **C{H} NMR** (101 MHz, CD_2_Cl_2_) *δ* = 164.4 (C=O), 159.0 (2 × DMT), 153.9 (C4), 152.1 (C2), 146.5 (C8), 145.4 (C6), 145.2 (DMT), 136.1 (2 × DMT), 133.4 (C5), 130.4 (2 × DMT), 128.4 (DMT), 128.2 (DMT), 127.2 (DMT), 113.5 (DMT), 87.2 (C4’), 86.8 (DMT), 85.3 (C1′), 72.1 (C3’), 64.4 (C5’), 55.6 (DMT), 53.3 (OCH_3_), 40.7 (C2’) ppm. ^**15**^ **N NMR** (41 MHz, CD_2_Cl_2_) *δ* = 276 (N1), 260 (N3), 242 (N7), 173 (N9) ppm. **ESI(+)‐MS m/z** [M + Na]^+^ calculated: 619.2163 found: 619.2173. Elemental analysis: C_33_H_32_N_4_O_7_ · 0.5 EtOAc: C 65.7; H 5.9; N 8.6; calcd. C 65.5; H 5.7; N 8.7.

**3′‐*O*‐[(2‐cyanoethoxy)(diisopropylamino)phosphino]‐5′‐*O*‐(4,4′‐dimethoxytrityl)‐*β*‐D‐2′‐deoxyribosylpurine‐6‐carboxylic acid methyl ester (6).** 5′‐*O*‐(4,4′‐dimethoxytrityl)‐*β*‐D‐2′‐deoxyribosylpurine‐6‐carboxylic acid methyl ester (0.40 g, 0.67 mmol, 1.0 eq.) and DIPEA (0.24 g, 1.8 mmol, 2.7 eq.) were dissolved in dry DCM (40 mL) and cooled to 0 °C. Subsequently, 2‐cyanoethyl‐*N*,*N*‐diisopropylchlorophosphoramidite (0.16 g, 0.70 mmol, 1.0 eq.) was added, and after warming the mixture to room temperature, it was stirred for 2 h. The solution was diluted with DCM and washed once with a saturated aqueous solution of NaHCO_3_ (50 mL). The organic layer was dried (MgSO_4_), and the solvent was removed in vacuo. The subsequent purification by column chromatography (CH:EtOAc:DCM:NEt_3_ 1:0:1:0.02 to 1:3:1:0.05) to give the product as a mixture of two diastereomers as a white foam (0.43 g, 0.55 mmol, 82%). Characterization of isomer **A**. ^**1**^ **H NMR** (500 MHz, CD_2_Cl_2_) *δ* = 8.97 (s, 1H, H2), 8.43 (s, 1H, H8), 7.45–7.37 (m, 2H, DMT), 7.31–7.28 (m, 4H, DMT), 7.28–7.19 (m, 3H, DMT), 6.85–6.75 (m, 4H, DMT), 6.54 (t, ^3^ *J*_HH_ = 6.5 Hz, 1H, H1′), 4.87–4.77 (m, 1H, H3’), 4.38–4.33 (m, 1H, H4’), 4.06 (s, 3H, OCH_3_), 3.77 (s, 6H, 2 × OCH_3_ (DMT)), 3.75–3.67 (m, 2H, P‐OCH_2_), 3.67–3.59 (m, 2H, ^*i*^Pr‐CH), 3.45–3.35 (m, 1H, H5’ or H5″), 3.35–3.25 (m, 1H, H5’ or H5″), 3.00–2.92(m, 1H, H2’ or H2″), 2.72–2.61 (m, 1H, H2’ or H2″), 2.51 (t, ^3^ *J*_HH_ = 6.8 Hz, 2H, CH_2_CN), 1.21 (d, ^3^ *J*_HH_ = 6.7 Hz, 6H, 2 × ^*i*^Pr‐CH_3_), 1.20 (d, ^3^ *J*_HH_ = 6.8 Hz, 6H, 2 × ^*i*^Pr‐CH_3_) ppm. ^**13**^ **C{H} NMR** (126 MHz, CD_2_Cl_2_) *δ* = 164.4 (C=O), 159.1 (DMT), 153.9 (C4), 152.2 (C2), 146.4 (C8), 145.7 (C6), 145.1 (DMT), 136.1 (DMT), 136.0 (DMT), 133.5 (C5), 130.4 (2 × DMT), 128.5 (DMT), 128.2 (DMT), 127.3 (DMT), 118.0 (CN), 113.5 (DMT), 86.9 (DMT), 86.5 (d, ^3^ *J*_CP_ = 7.0 Hz, C4’), 85.3 (C1′), 73.8 (d, ^2^ *J*_CP_ = 16.9 Hz, C3’), 63.8 (C5’), 58.8 (d, ^2^ *J*_CP_ = 19.0 Hz, P‐OCH_2_), 55.6 (OCH_3_ (DMT)), 53.3 (OCH_3_), 43.7 (d, ^2^ *J*_CP_ = 12.4 Hz, ^*i*^Pr‐CH), 39.7 (d, ^3^ *J*_CP_ = 4.4 Hz, C2’), 24.8 (d, ^3^ *J*_CP_ = 7.0 Hz, ^*i*^Pr‐CH_3_), 20.7 (d, ^3^ *J*_CP_ = 7.0 Hz, CH_2_CN) ppm. ^**15**^ **N NMR** (51 MHz, CD_2_Cl_2_) *δ* = 277 (N1), 260 (N3), 249 (CN), 243 (N7), 173 (N9), 100 (^*i*^Pr‐N) ppm. ^**31**^ **P{H} NMR** (202 MHz, CD_2_Cl_2_) *δ* = 148.82 ppm. Characterization of isomer **B**. ^**1**^ **H NMR** (500 MHz, CD_2_Cl_2_) *δ* = 8.96 (s, 1H, H2), 8.41 (s, 1H, H8), 7.42–7.37 (m, 2H, DMT), 7.29–7.27 (m, 4H, DMT), 7.25–7.18 (m, 3H, DMT), 6.83–6.75 (m, 4H, DMT), 6.54 (t, ^3^ *J*_HH_ = 6.5 Hz, 1H, H1′), 4.85–4.75 (m, 1H, H3’), 4.34–4.28 (m, 1H, H4’), (s, 3H, OCH_3_), 3.77 (s, 6H, 2 × OCH_3_ (DMT)), 3.80–3.73 (m, 2H, P‐OCH_2_), 3.62 (m, 2H, ^*i*^Pr‐CH), 3.41–3.32 (m, 2H, H5’, H5″), 3.00–2.91 (m, 1H, H2’ or H2″), 2.77–2.69 (m, 1H, H2’ or H2″), 2.63 (t, ^3^ *J*_HH_ = 6.3 Hz, 2H, CH_2_CN), 1.20 (d, ^3^ *J*_HH_ = 6.8 Hz, 6H, 2 × ^*i*^Pr‐CH3), 1.14 (d, ^3^ *J*_HH_ = 6.8 Hz, 6H, 2 × ^*i*^Pr‐CH_3_) ppm. ^**13**^ **C{H} NMR** (126 MHz, CD_2_Cl_2_) *δ* = 164.4 (C=O), 159.1 (DMT), 153.9 (C4), 152.2 (C2), 146.4 (C8), 145.7 (C6), 145.1 (DMT), 136.1 (DMT), 136.0 (DMT), 133.5 (C5), 130.4 (2 × DMT), 128.4 (DMT), 128.2 (DMT), 127.2 (DMT), 118.2 (CN), 113.5 (DMT), 86.9 (DMT), 86.3(d, ^3^ *J*_CP_ = 6.2 Hz, C4’), 85.3 (C1′), 74.4 (d, ^2^ *J*_CP_ = 17.3 Hz, C3’), 63.9 (C5’), 58.7 (d, ^2^ *J*_CP_ = 19.3 Hz, P‐OCH_2_), 55.6 (OCH_3_ (DMT)), 53.3 (OCH_3_), 43.7 (d, ^2^ *J*_CP_ = 12.4 Hz, ^*i*^Pr‐CH), 39.7 (d, ^3^ *J*_CP_ = 3.2 Hz, C2’), 24.7 (d, ^3^ *J*_CP_ = 7.0 Hz, ^*i*^Pr‐CH_3_), 20.8 (d, ^3^ *J*_CP_ = 7.2 Hz, CH_2_CN) ppm. ^**15**^ **N NMR** (51 MHz, CD_2_Cl_2_) *δ* = 277 (N1), 260 (N3), 248 (CN), 243 (N7), 173 (N9), 99 (^*i*^Pr‐N) ppm. ^**31**^ **P{H} NMR** (202 MHz, CD_2_Cl_2_) *δ* = 148.83 ppm. Characterization of a mixture of **A** and **B**. ESI(+)‐MS m/z [M + H]^+^ calculated: 797.3422; found: 797.3410. Elemental analysis: C_42_H_49_N_6_O_8_P · 0.2 CH_2_Cl_2_: C 62.4; H 6.4; N 10.2; calcd. C 62.3; H 6.1; N 10.3.

## Conflict of Interest

The authors declare no conflict of interest.

## Supporting information

Supplementary Material

## Data Availability

The data that support the findings of this study are available from the corresponding author upon reasonable request.
