# Supplementary material for: Electrochemical Characterization of Site‐Specifically Metal‐Modified DNA Films on Gold Electrode Surfaces
Source: Chempluschem. 2025 Oct 9;90(12):e202500494. doi: 10.1002/cplu.202500494 (PMC12701301; doi:10.1002/cplu.202500494)
Supplement: Supplementary file 1 — Supplementary Material [file CPLU-90-e202500494-s001.pdf]

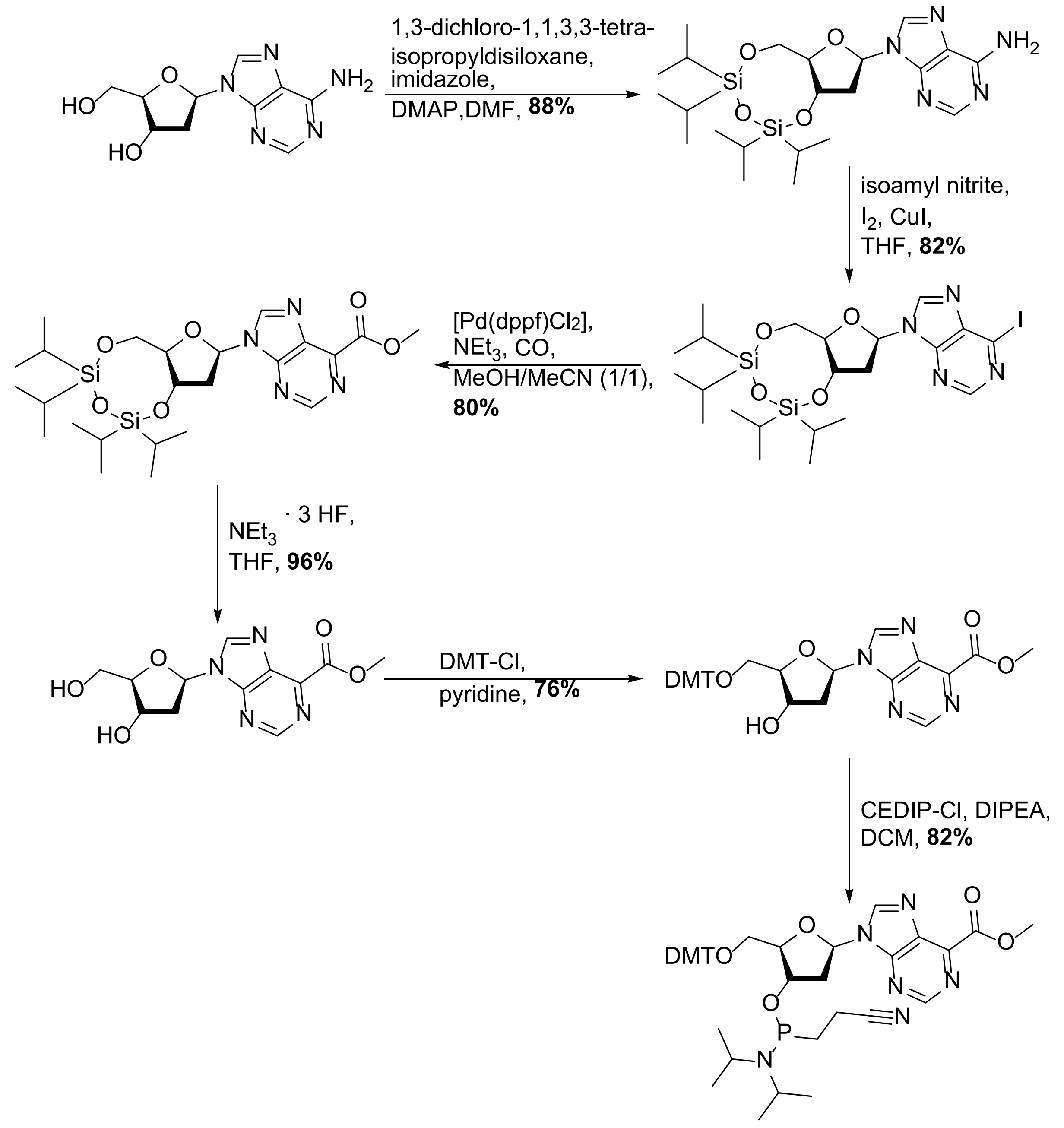

Scheme S1: Synthesis scheme of 3′-*O*-[(2-cyanoethoxy)(diisopropylamino)phosphino]-5′-*O*-(4,4′-dimethoxytrityl)-β-D-2′-deoxyribosylpurine-6-carboxylic acid methyl ester as optimized in this work.[1]

Table S1: Summary of the 26mer oligonucleotide duplexes used in this study with highlighted metal metal-binding site and linker (**H** = hydroxypyridone, **K** = imidazole carboxylate, **P** = purine carboxylate, **D** = 7-deaza-6-pyrazolylpurine, R = –$(CH_2)_6S_2(CH_2)_6OH$).

Duplex	Sequences
**HH**$_{C}$	R-5'-d(TTT G**H**T TGT TTG TTT GTT TTT TTT TT)-3'<br>3'-d(AAA C**H**A ACA AAC AAA CAA AAA AAA AA)-5'
**HK**$_{C}$	R-5'-d(TTT G**H**T TGT TTG TTT GTT TTT TTT TT)-3'<br>3'-d(AAA C**K**A ACA AAC AAA CAA AAA AAA AA)-5'
**HP**$_{C}$	R-5'-d(TTT G**H**T TGT TTG TTT GTT TTT TTT TT)-3'<br>3'-d(AAA C**P**A ACA AAC AAA CAA AAA AAA AA)-5'
**PH**$_{C}$	R-5'-d(TTT G**P**T TGT TTG TTT GTT TTT TTT TT)-3'<br>3'-d(AAA C**H**A ACA AAC AAA CAA AAA AAA AA)-5'
**PP**$_{C}$	R-5'-d(TTT G**P**T TGT TTG TTT GTT TTT TTT TT)-3'<br>3'-d(AAA C**P**A ACA AAC AAA CAA AAA AAA AA)-5'
**DD**$_{C}$	R-5'-d(TTT G**D**T TGT TTG TTT GTT TTT TTT TT)-3'<br>3'-d(AAA C**D**A ACA AAC AAA CAA AAA AAA AA)-5'
**CD**$_{C}$	R-5'-d(TTT G**C**T TGT TTG TTT GTT TTT TTT TT)-3'<br>3'-d(AAA C**D**A ACA AAC AAA CAA AAA AAA AA)-5'
**HH**$_{M}$	R-5'-d(TTT GTT TGT TTG T**H**T GTT TTT TTT TT)-3'<br>3'-d(AAA CAA ACA AAC A**H**A CAA AAA AAA AA)-5'
**HK**$_{M}$	R-5'-d(TTT GTT TGT TTG T**H**T GTT TTT TTT TT)-3'<br>3'-d(AAA CAA ACA AAC A**K**A CAA AAA AAA AA)-5'
**HP**$_{M}$	R-5'-d(TTT GTT TGT TTG T**H**T GTT TTT TTT TT)-3'<br>3'-d(AAA CAA ACA AAC A**P**A CAA AAA AAA AA)-5'
**PH**$_{M}$	R-5'-d(TTT GTT TGT TTG T**P**T GTT TTT TTT TT)-3'<br>3'-d(AAA CAA ACA AAC A**H**A CAA AAA AAA AA)-5'
**PP**$_{M}$	R-5'-d(TTT GTT TGT TTG T**P**T GTT TTT TTT TT)-3'<br>3'-d(AAA CAA ACA AAC A**P**A CAA AAA AAA AA)-5'
**DD**$_{M}$	R-5'-d(TTT GTT TGT TTG T**D**T GTT TTT TTT TT)-3'<br>3'-d(AAA CAA ACA AAC A**D**A CAA AAA AAA AA)-5'
**CD**$_{M}$	R-5'-d(TTT GTT TGT TTG T**C**T GTT TTT TTT TT)-3'<br>3'-d(AAA CAA ACA AAC A**D**A CAA AAA AAA AA)-5'
**HH**$_{F}$	R-5'-d(TTT GTT TGT TTG TTT GTT TTT **H**TT TT)-3'<br>3'-d(AAA CAA ACA AAC AAA CAA AAA **H**AA AA)-5'
**HK**$_{F}$	R-5'-d(TTT GTT TGT TTG TTT GTT TTT **H**TT TT)-3'<br>3'-d(AAA CAA ACA AAC AAA CAA AAA **K**AA AA)-5'
**HP**$_{F}$	R-5'-d(TTT GTT TGT TTG TTT GTT TTT **H**TT TT)-3'<br>3'-d(AAA CAA ACA AAC AAA CAA AAA **P**AA AA)-5'

Duplex	Sequences
**PH$_F$**	R-5'-d(TTT GTT TGT TTG TTT GTT TTT **P**TT TT)-3'<br>3'-d(AAA CAA ACA AAC AAA CAA AAA **H**AA AA)-5'
**PP$_F$**	R-5'-d(TTT GTT TGT TTG TTT GTT TTT **P**TT TT)-3'<br>3'-d(AAA CAA ACA AAC AAA CAA AAA **P**AA AA)-5'
**DD$_F$**	R-5'-d(TTT GTT TGT TTG TTT GTT TTT **D**TT TT)-3'<br>3'-d(AAA CAA ACA AAC AAA CAA AAA **D**AA AA)-5'
**CD$_F$**	R-5'-d(TTT GTT TGT TTG TTT GTT TTT **C**TT TT)-3'<br>3'-d(AAA CAA ACA AAC AAA CAA AAA **D**AA AA)-5'

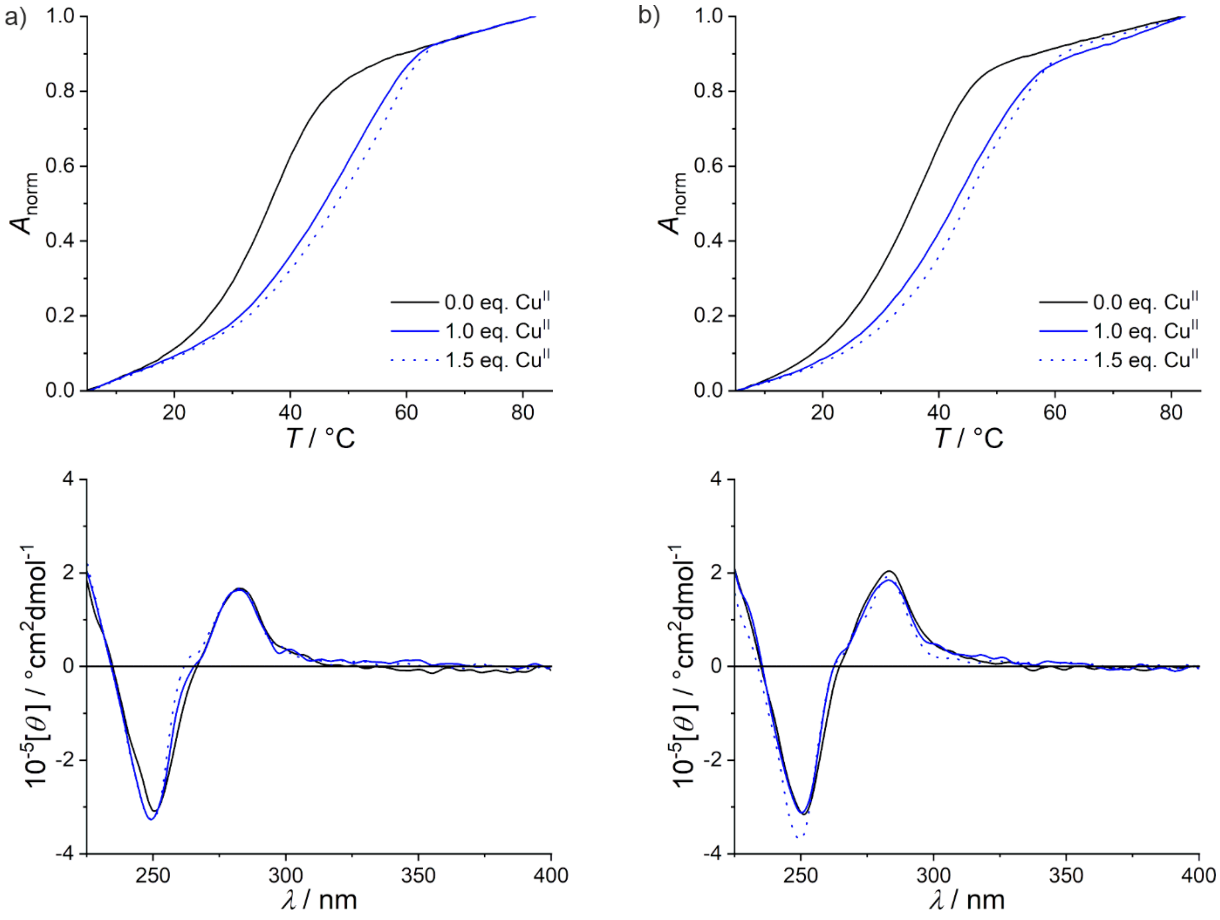

Figure S1: Melting curves (top) and CD spectra (bottom) of duplexes a) $\mathbf{PP_M}$ and b) $\mathbf{PP_F}$ in the presence of increasing amounts of Cu^{II}. Experimental conditions: 1 μM dsDNA, 5 mM MOPS (pH 6.8), 150 mM $NaClO_4$, 1.0 eq. Cu^{II} ≙ 1 μM $Cu(NO_3)_2$.

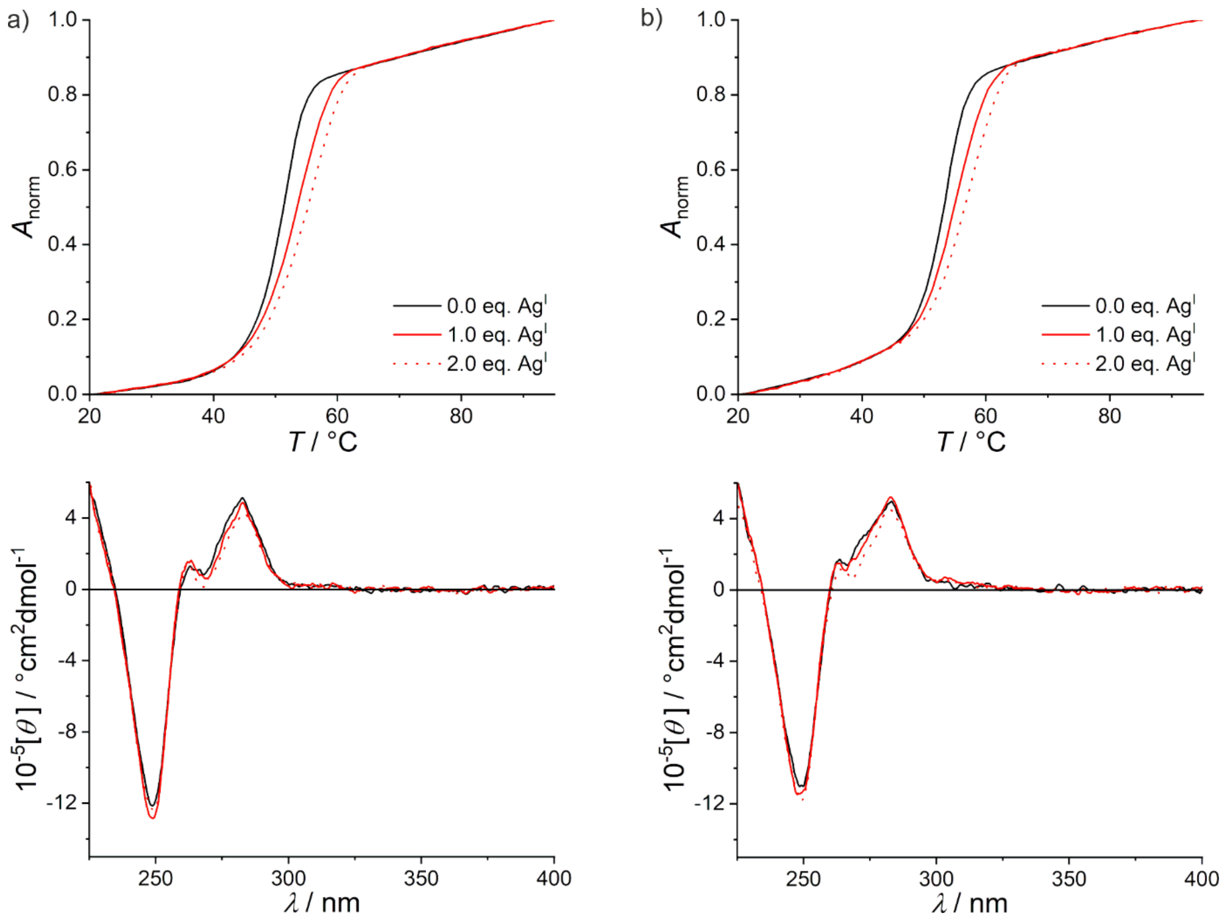

Figure S2: Melting curves (top) and CD spectra (bottom) of duplexes a) **CD$_M$** and b) **CD$_F$** in the presence of increasing amounts of Ag^I. Experimental conditions: 1 μM dsDNA, 5 mM MOPS (pH 6.8), 150 mM $NaClO_4$, 1.0 eq. Ag^I ≙ 1 μM $AgNO_3$.

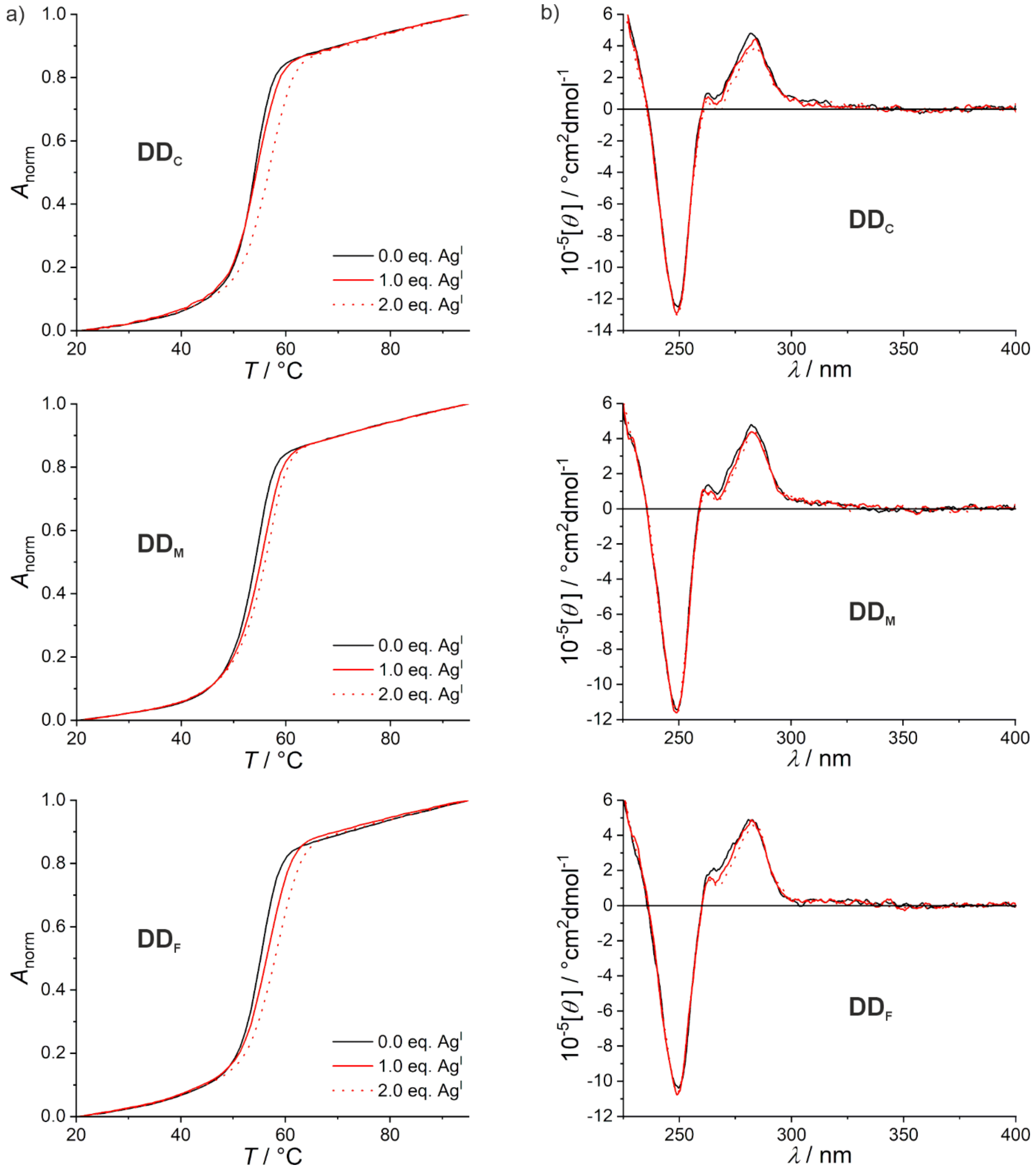

Figure S3: a) Melting curves and b) CD spectra of duplexes **DD$_C$** (top), **DD$_M$** (center), and **DD$_F$** (bottom) in the presence of increasing amounts of Ag^I. Experimental conditions: 1 μM dsDNA, 5 mM MOPS (pH 6.8), 150 mM $NaClO_4$, 1.0 eq. Ag^I ≙ 1 μM $AgNO_3$.

Table S2: Melting temperatures in the absence ($T_{m,0}$) and presence of 1.0 eq. of Cu^{II} or Ag^{I} ($T_{m,1}$) as well as increase in T_m ($\Delta T_{m,0\rightarrow1}$).

Duplex	$T_{m,0}$ / °C	$T_{m,1}$ / °C	$T_{m,0\rightarrow1}$ / °C
$\mathbf{PP_C}$	37	42	5
$\mathbf{DD_C}$	54	54	0
$\mathbf{CD_C}$	52	55	3
$\mathbf{PP_M}$	36	47	11
$\mathbf{DD_M}$	54	55	1
$\mathbf{CD_M}$	51	53	2
$\mathbf{PP_F}$	35	43	8
$\mathbf{DD_F}$	55	56	1
$\mathbf{CD_F}$	54	56	2

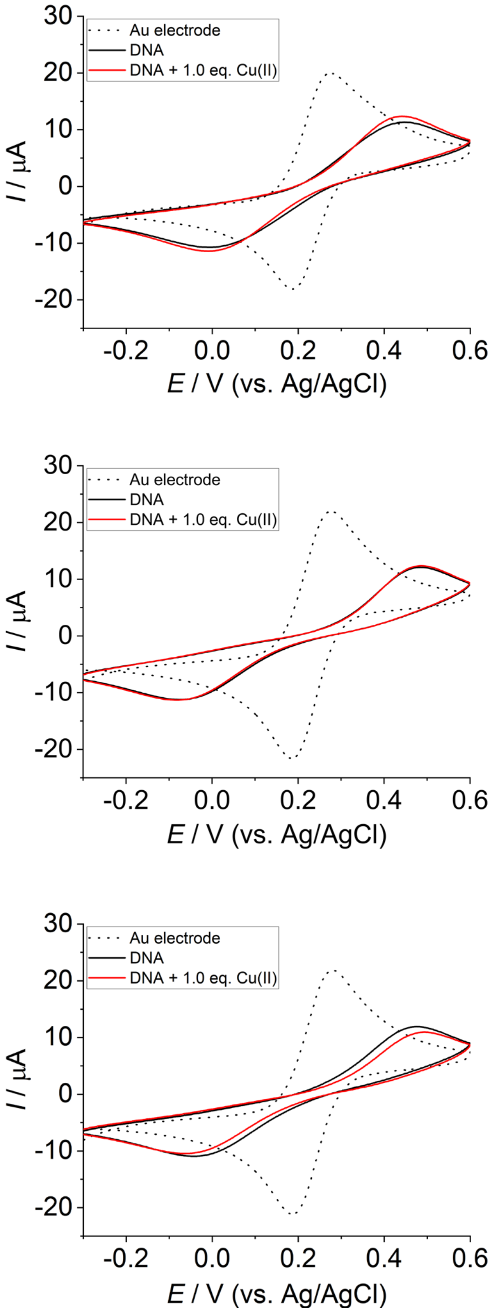

Figure S4: Representative cyclic voltammograms of Au electrodes with and without DNA films of **HH$_{C}$** (top), **HH$_{M}$** (center), and **HH$_{F}$** (bottom). Experimental conditions: 2 mM $K_4[Fe(CN)_6]$, 2 mM $K_3[Fe(CN)_6]$, 5 mM MOPS (pH 6.8), 150 mM $NaClO_4$.

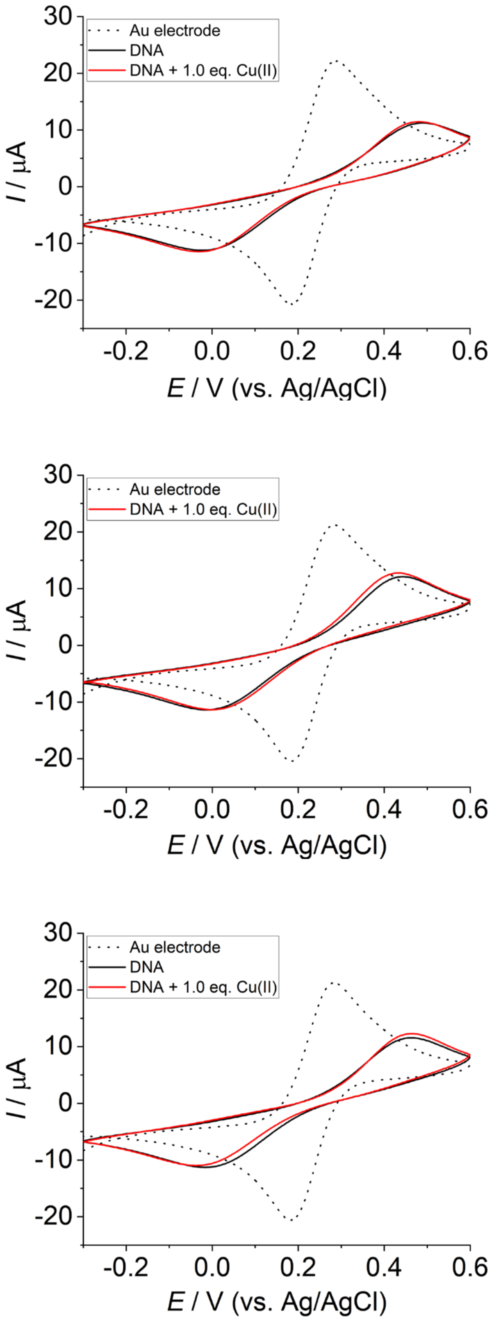

Figure S5: Representative cyclic voltammograms of Au electrodes with and without DNA films of **HK**$_{\mathbf{C}}$ (top), **HK**$_{\mathbf{M}}$ (center), and **HK**$_{\mathbf{F}}$ (bottom). Experimental conditions: 2 mM $K_4[Fe(CN)_6]$, 2 mM $K_3[Fe(CN)_6]$, 5 mM MOPS (pH 6.8), 150 mM $NaClO_4$.

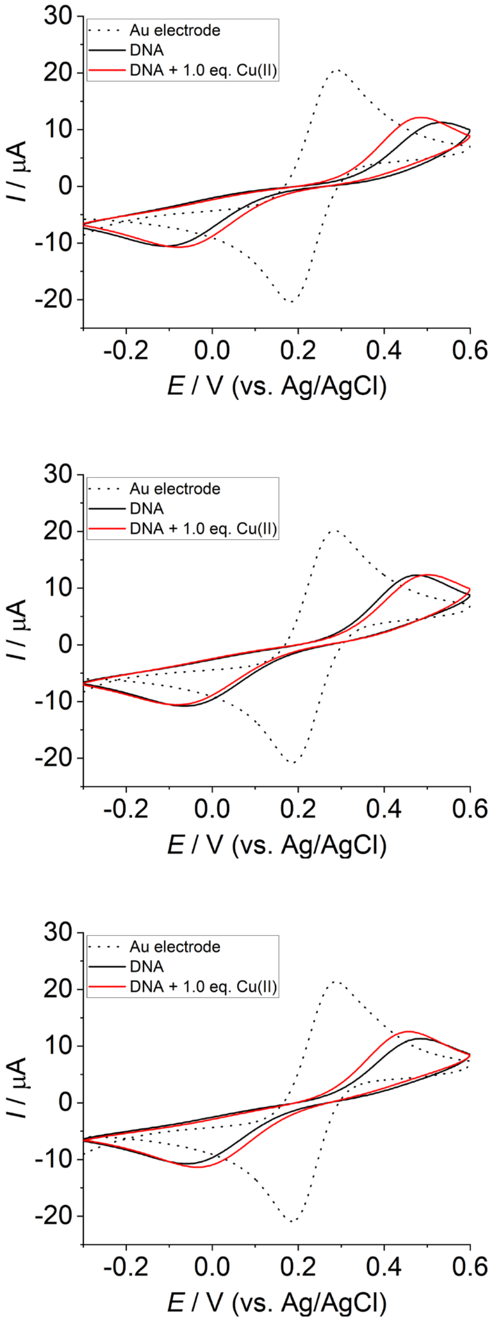

Figure S6: Representative cyclic voltammograms of Au electrodes with and without DNA films of **HP$_C$** (top), **HP$_M$** (center), and **HP$_F$** (bottom). Experimental conditions: 2 mM $K_4[Fe(CN)_6]$, 2 mM $K_3[Fe(CN)_6]$, 5 mM MOPS (pH 6.8), 150 mM $NaClO_4$.

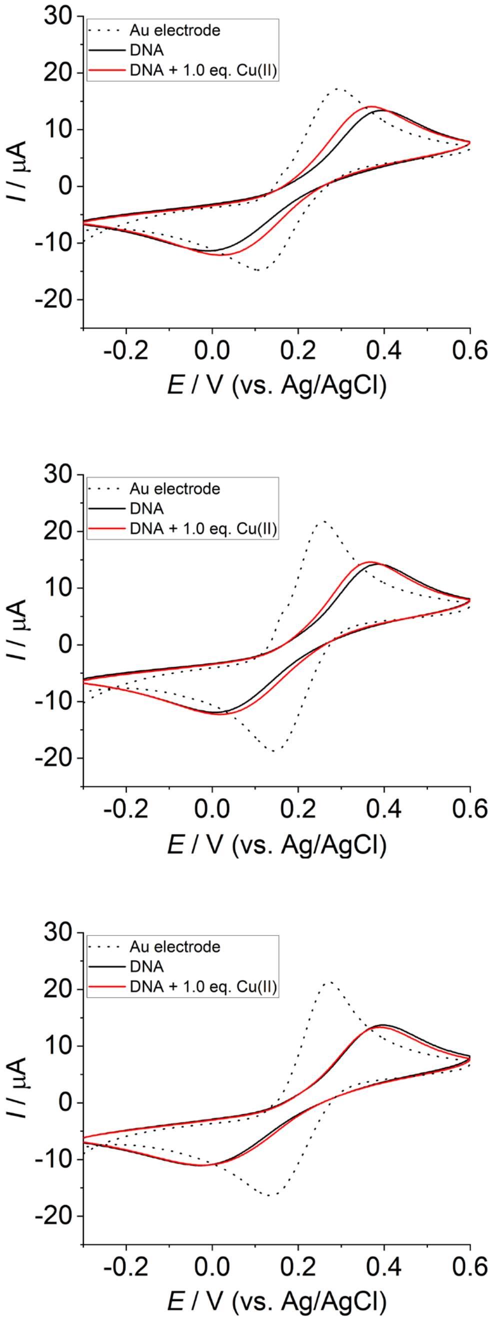

Figure S7: Representative cyclic voltammograms of Au electrodes with and without DNA films of **PH$_C$** (top), **PH$_M$** (center), and **PH$_F$** (bottom). Experimental conditions: 2 mM $K_4[Fe(CN)_6]$, 2 mM $K_3[Fe(CN)_6]$, 5 mM MOPS (pH 6.8), 150 mM $NaClO_4$.

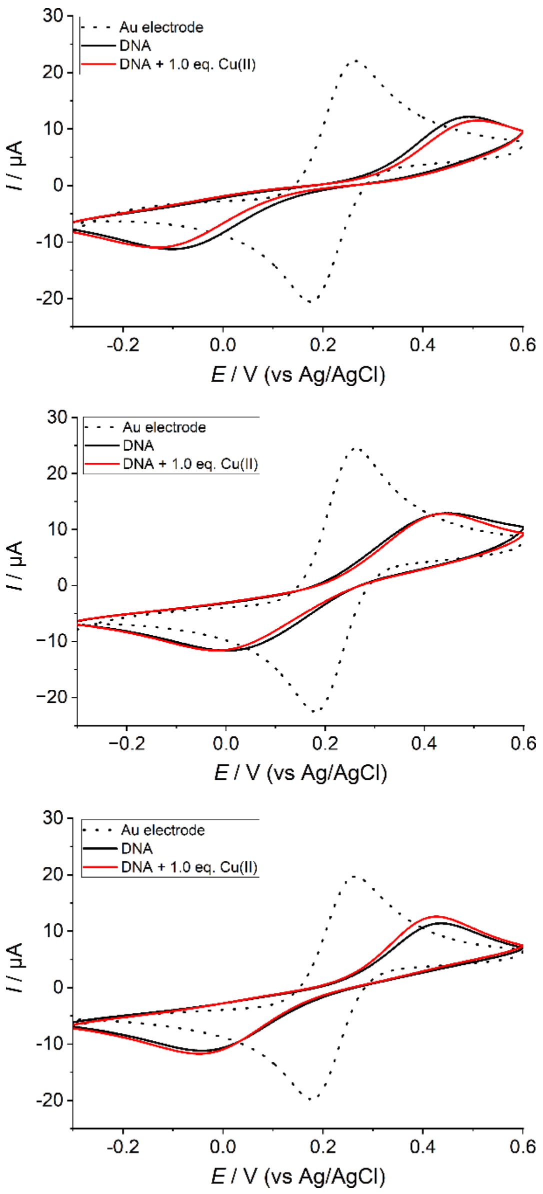

Figure S8: Representative cyclic voltammograms of Au electrodes with and without DNA films of $\mathbf{PP_C}$ (top), $\mathbf{PP_M}$ (middle), and $\mathbf{PP_F}$ (bottom). Experimental conditions: 2 mM $K_4[Fe(CN)_6]$, 2 mM $K_3[Fe(CN)_6]$, 5 mM MOPS (pH 6.8), 150 mM $NaClO_4$.

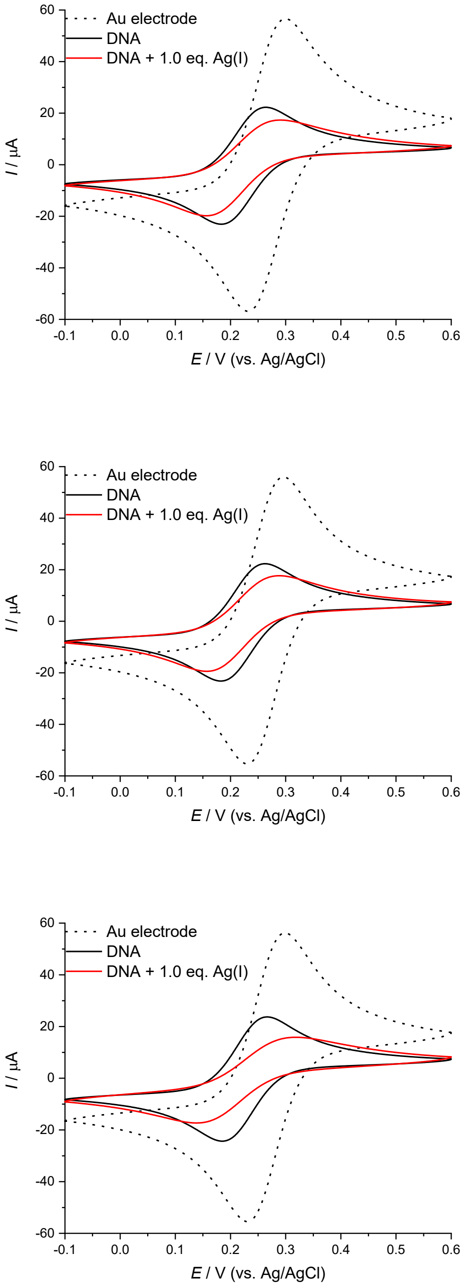

Figure S9: Representative cyclic voltammograms of Au electrodes with and without DNA films of $\mathbf{DD_C}$ (top), $\mathbf{DD_M}$ (middle), and $\mathbf{DD_F}$ (bottom). Experimental conditions: 2 mM $K_4[Fe(CN)_6]$, 2 mM $K_3[Fe(CN)_6]$, 5 mM MOPS (pH 6.8), 150 mM $NaClO_4$.

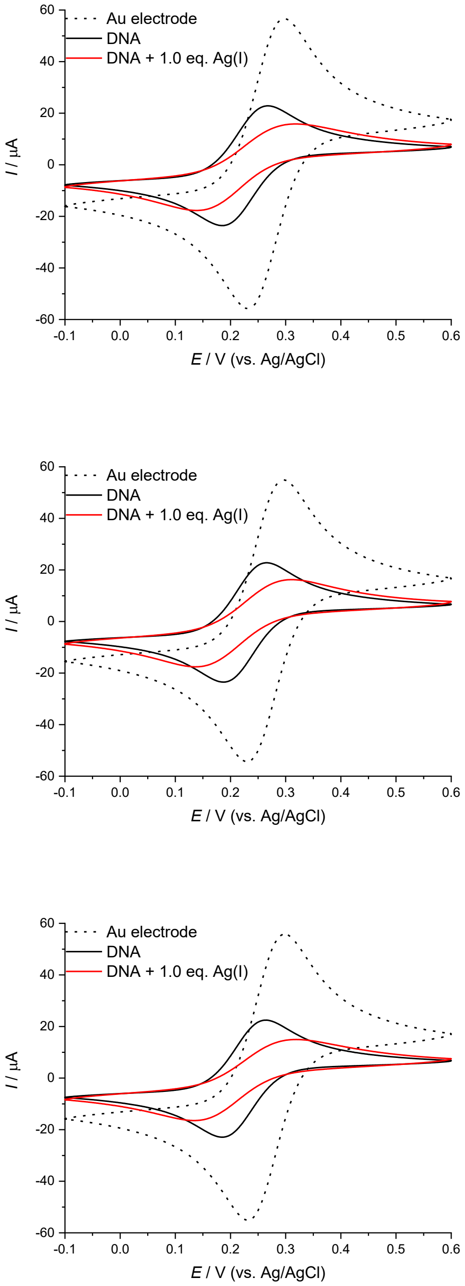

Figure S10: Representative cyclic voltammograms of Au electrodes with and without DNA films of **CD_C** (top), **CD_M** (middle), and **CD_F** (bottom). Experimental conditions: 2 mM $K_4[Fe(CN)_6]$, 2 mM $K_3[Fe(CN)_6]$, 5 mM MOPS (pH 6.8), 150 mM $NaClO_4$.

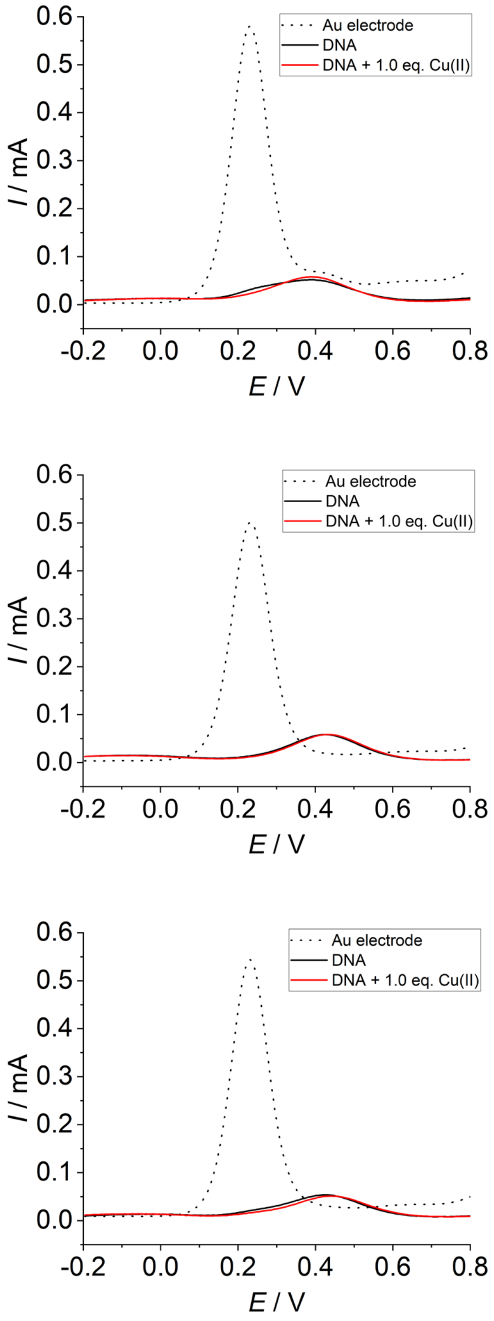

Figure S11: Representative square-wave voltammograms of Au electrodes with and without DNA films of **HH$_C$** (top), **HH$_M$** (center), and **HH$_F$** (bottom). Experimental conditions: 2 mM $K_4[Fe(CN)_6]$, 2 mM $K_3[Fe(CN)_6]$, 5 mM MOPS (pH 6.8), 150 mM $NaClO_4$.

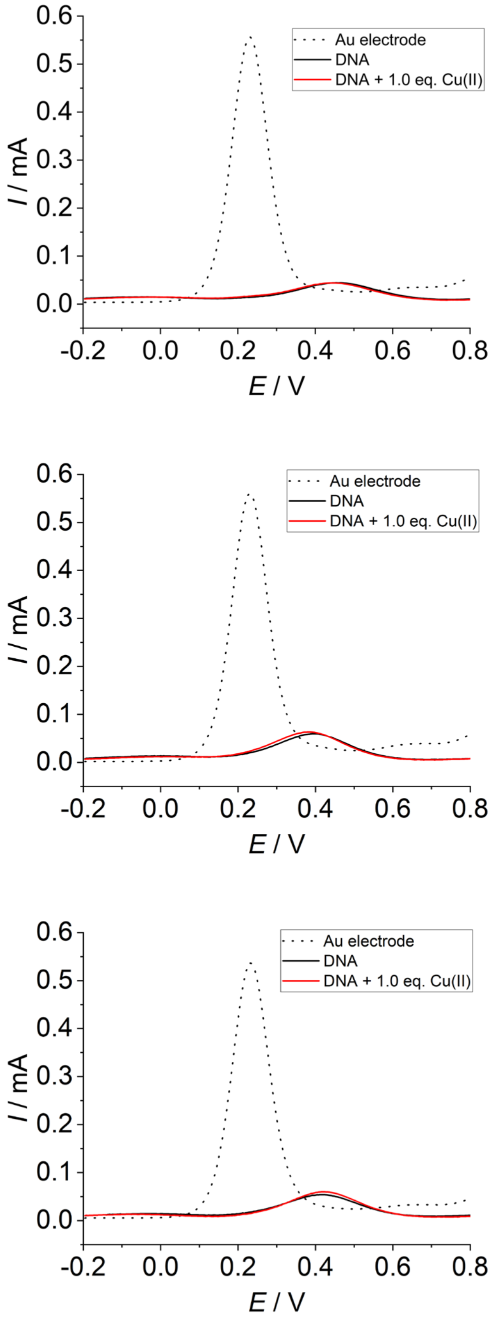

Figure S12: Representative square-wave voltammograms of Au electrodes with and without DNA films of **HK$_C$** (top), **HK$_M$** (center), and **HK$_F$** (bottom). Experimental conditions: 2 mM $K_4[Fe(CN)_6]$, 2 mM $K_3[Fe(CN)_6]$, 5 mM MOPS (pH 6.8), 150 mM $NaClO_4$.

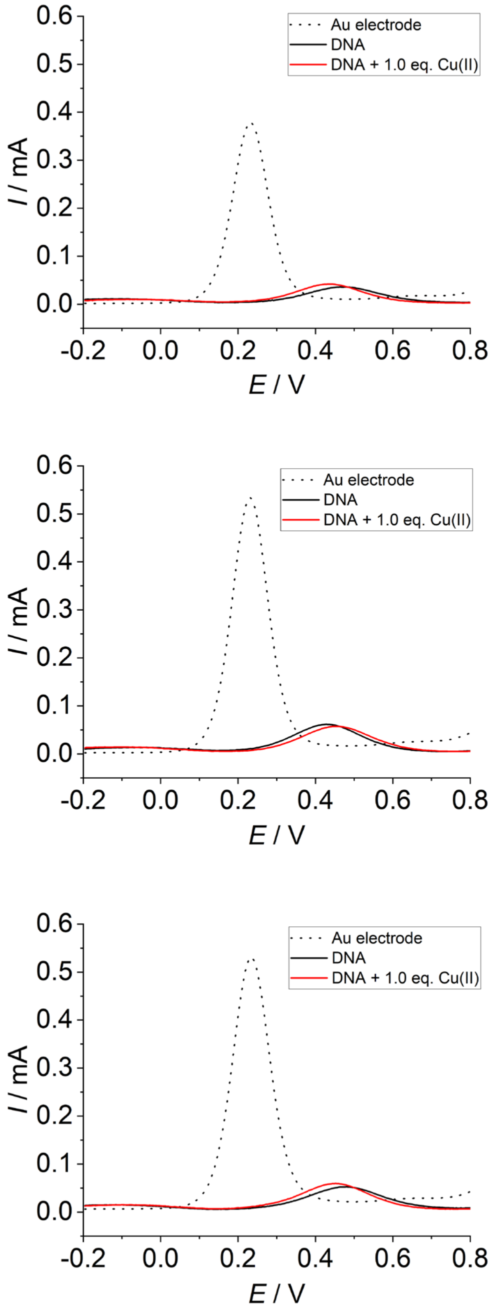

Figure S13: Representative square-wave voltammograms of Au electrodes with and without DNA films of **HP$_C$** (top), **HP$_M$** (center), and **HP$_F$** (bottom). Experimental conditions: 2 mM $K_4[Fe(CN)_6]$, 2 mM $K_3[Fe(CN)_6]$, 5 mM MOPS (pH 6.8), 150 mM $NaClO_4$.

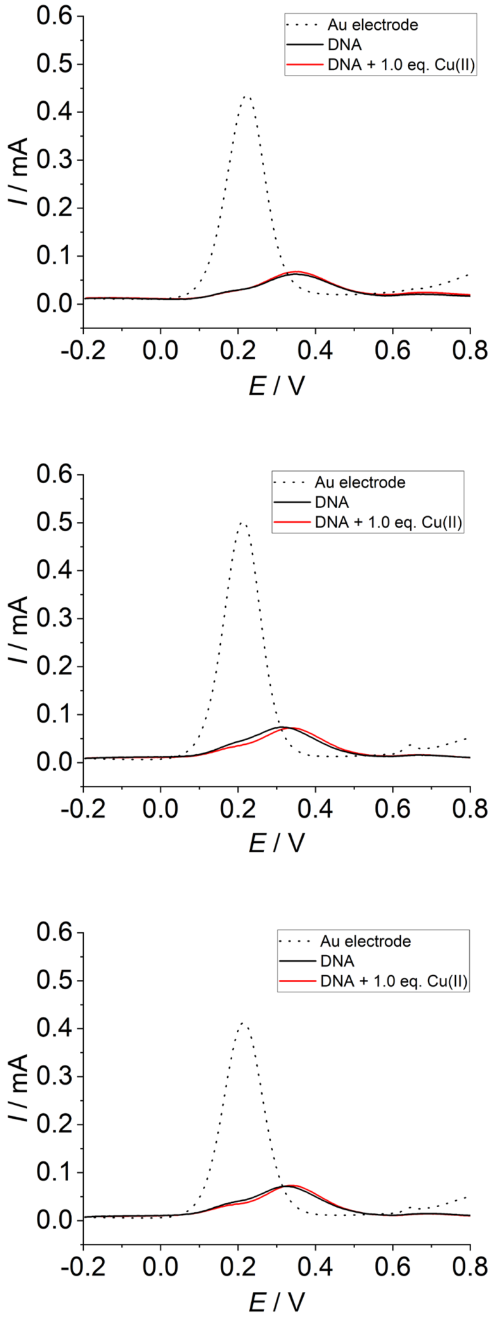

Figure S14: Representative square-wave voltammograms of Au electrodes with and without DNA films of **PH**$_{\mathbf{C}}$ (top), **PH**$_{\mathbf{M}}$ (center), and **PH**$_{\mathbf{F}}$ (bottom). Experimental conditions: 2 mM $K_4[Fe(CN)_6]$, 2 mM $K_3[Fe(CN)_6]$, 5 mM MOPS (pH 6.8), 150 mM $NaClO_4$.

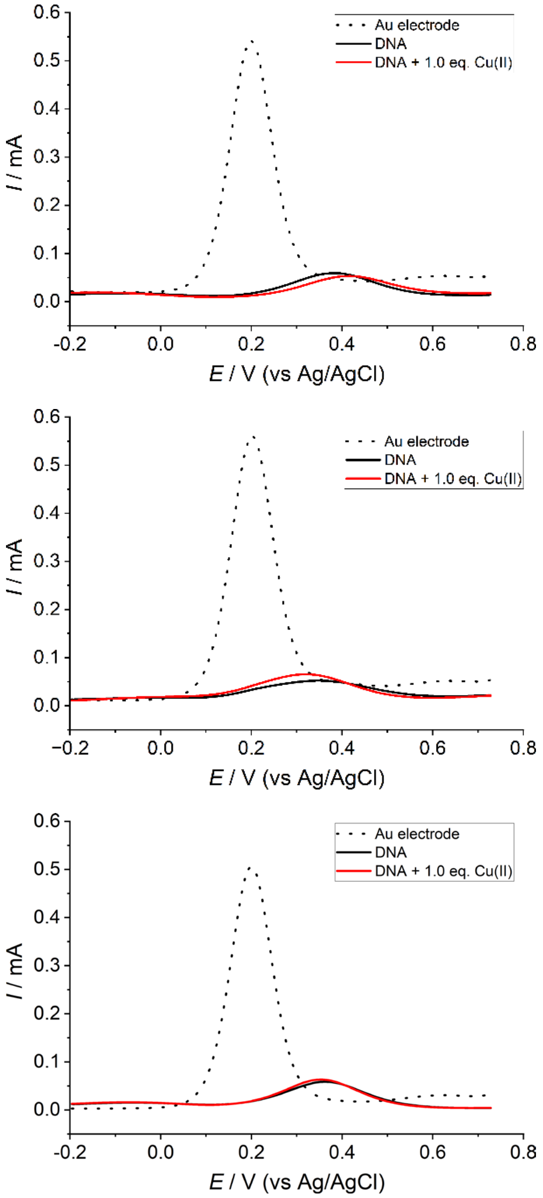

Figure S15: Representative square-wave voltammograms of Au electrodes with and without DNA films of **PP$_C$** (top), **PP$_M$** (center), and **PP$_F$** (bottom). Experimental conditions: 2 mM $K_4[Fe(CN)_6]$, 2 mM $K_3[Fe(CN)_6]$, 5 mM MOPS (pH 6.8), 150 mM $NaClO_4$.

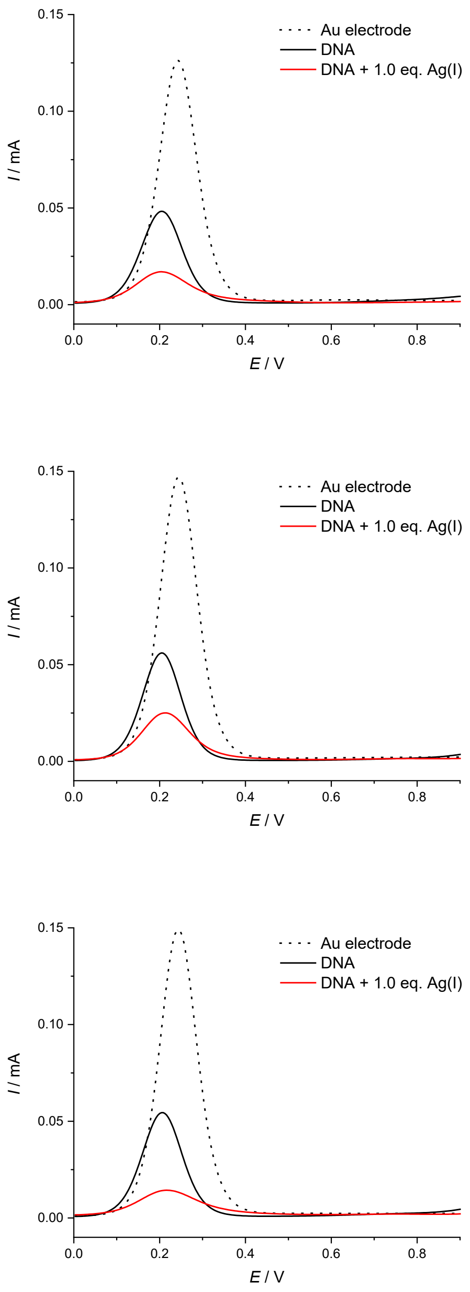

Figure S16: Representative square-wave voltammograms of Au electrodes with and without DNA films of **DD**$_{\mathbf{C}}$ (top), **DD**$_{\mathbf{M}}$ (center), and **DD**$_{\mathbf{F}}$ (bottom). Experimental conditions: 2 mM $K_4[Fe(CN)_6]$, 2 mM $K_3[Fe(CN)_6]$, 5 mM MOPS (pH 6.8), 150 mM $NaClO_4$.

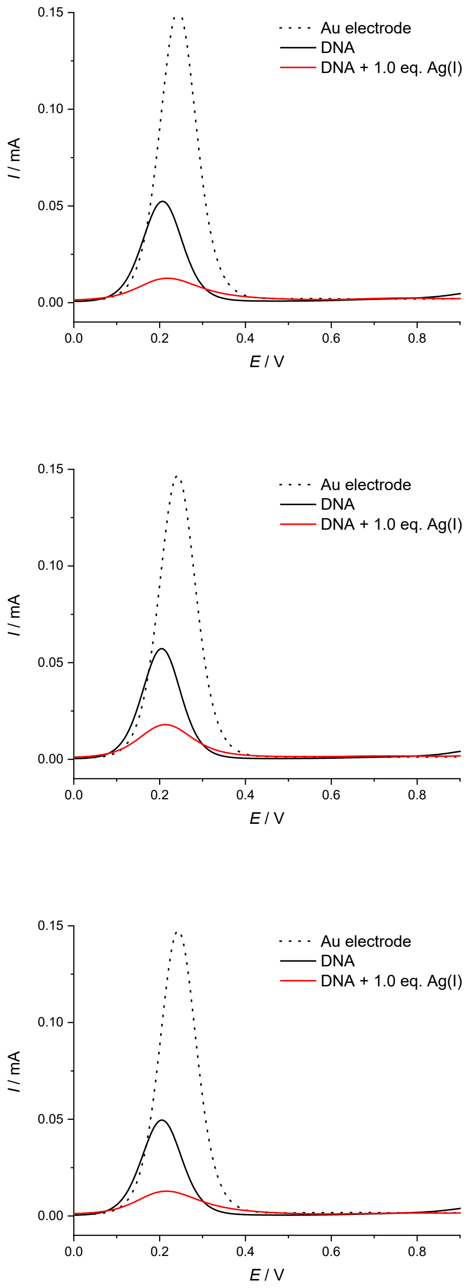

Figure S17: Representative square-wave voltammograms of Au electrodes with and without DNA films of **CD**$_{\mathbf{C}}$ (top), **CD**$_{\mathbf{M}}$ (center), and **CD**$_{\mathbf{F}}$ (bottom). Experimental conditions: 2 mM $K_4[Fe(CN)_6]$, 2 mM $K_3[Fe(CN)_6]$, 5 mM MOPS (pH 6.8), 150 mM $NaClO_4$.

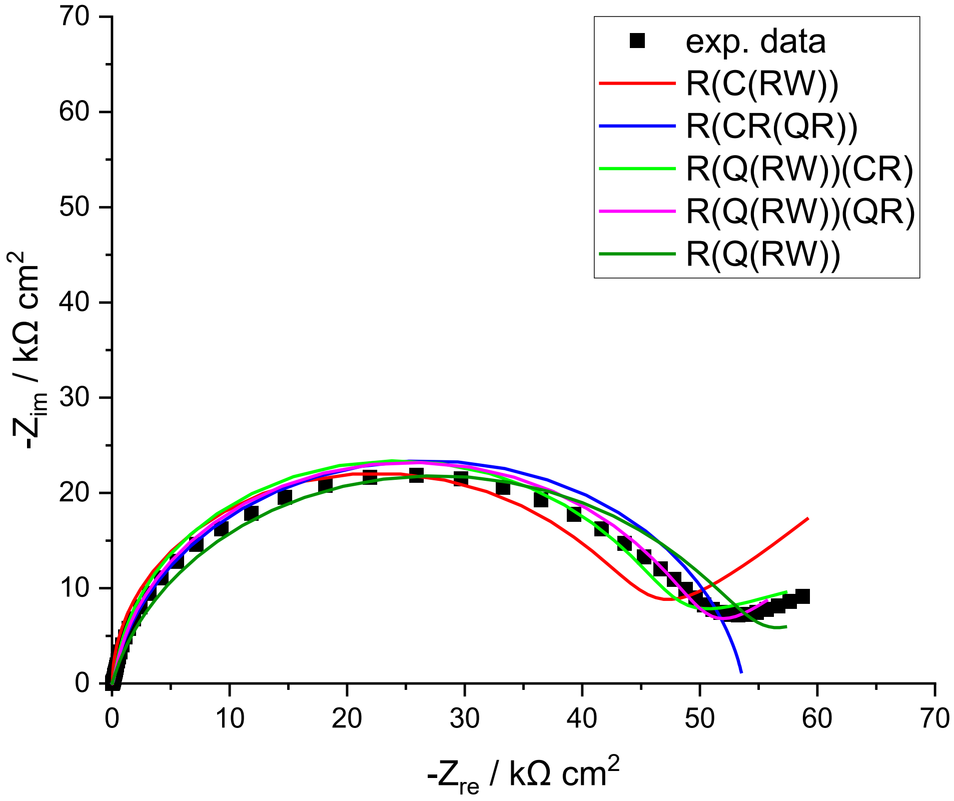

Figure S18: Comparison of different fits used for an analysis of the EIS data for all strands used within this work. Exemplarily shown is the Nyquist plot of the data of **HK_F**.

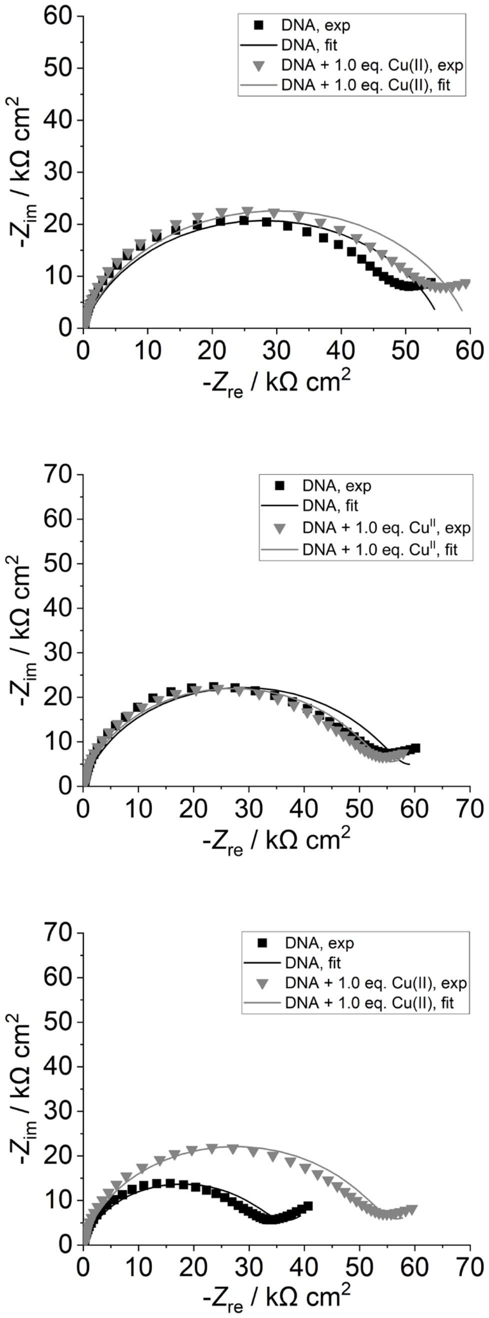

Figure S19: Representative Nyquist plot of the EIS data of a DNA film containing duplex **HH_C** (top), **HH_M** (center), and **HH_F** (bottom) immobilized on a gold electrode. The data were fitted using the modified Randles circuit shown in Figure 6. Experimental conditions: 2 mM $K_4[Fe(CN)_6]$, 2 mM $K_3[Fe(CN)_6]$, 5 mM MOPS (pH 6.8), 150 mM $NaClO_4$.

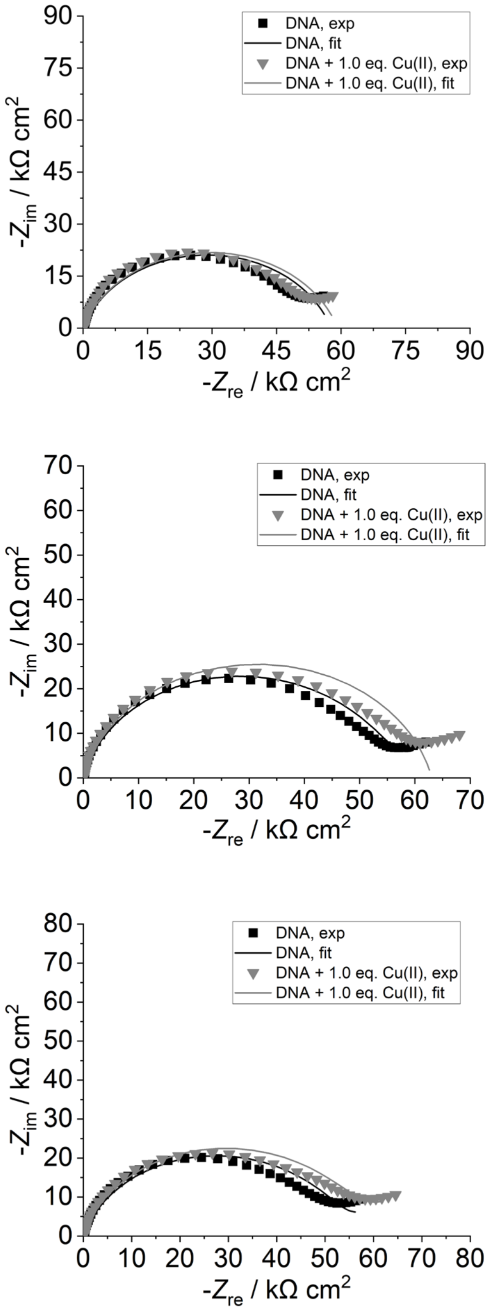

Figure S20: Representative Nyquist plot of the EIS data of a DNA film containing duplex **HK_C** (top), **HK_M** (center), and **HK_F** (bottom) immobilized on a gold electrode. The data were fitted using the modified Randles circuit shown in Figure 6. Experimental conditions: 2 mM $K_4[Fe(CN)_6]$, 2 mM $K_3[Fe(CN)_6]$, 5 mM MOPS (pH 6.8), 150 mM $NaClO_4$.

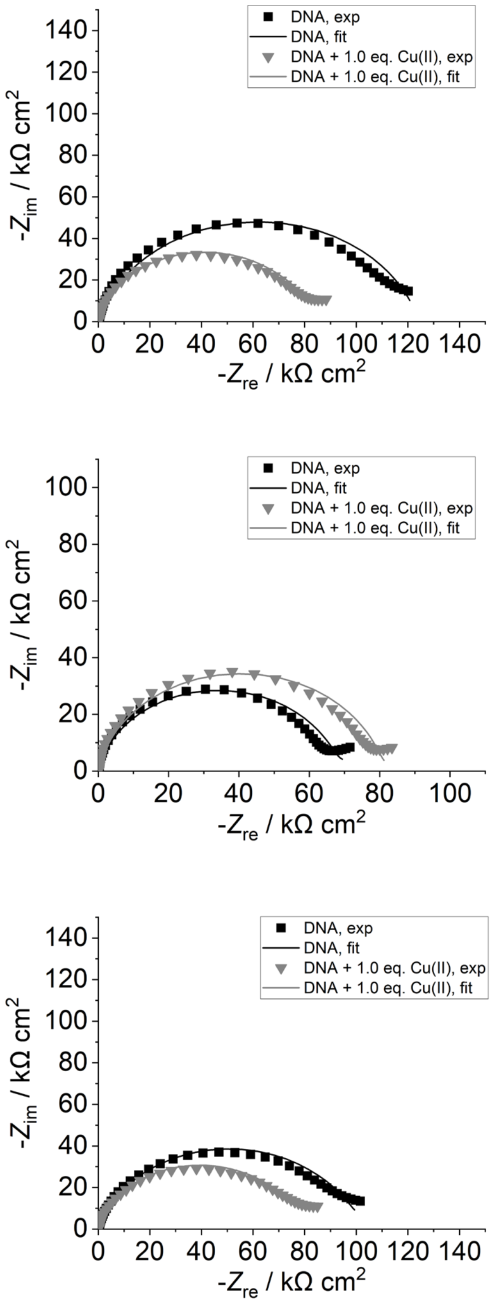

Figure S21: Representative Nyquist plot of the EIS data of a DNA film containing duplex **HP$_C$** (top), **HP$_M$** (center), and **HP$_F$** (bottom) immobilized on a gold electrode. The data were fitted using the modified Randles circuit shown in Figure 6. Experimental conditions: 2 mM $K_4[Fe(CN)_6]$, 2 mM $K_3[Fe(CN)_6]$, 5 mM MOPS (pH 6.8), 150 mM $NaClO_4$.

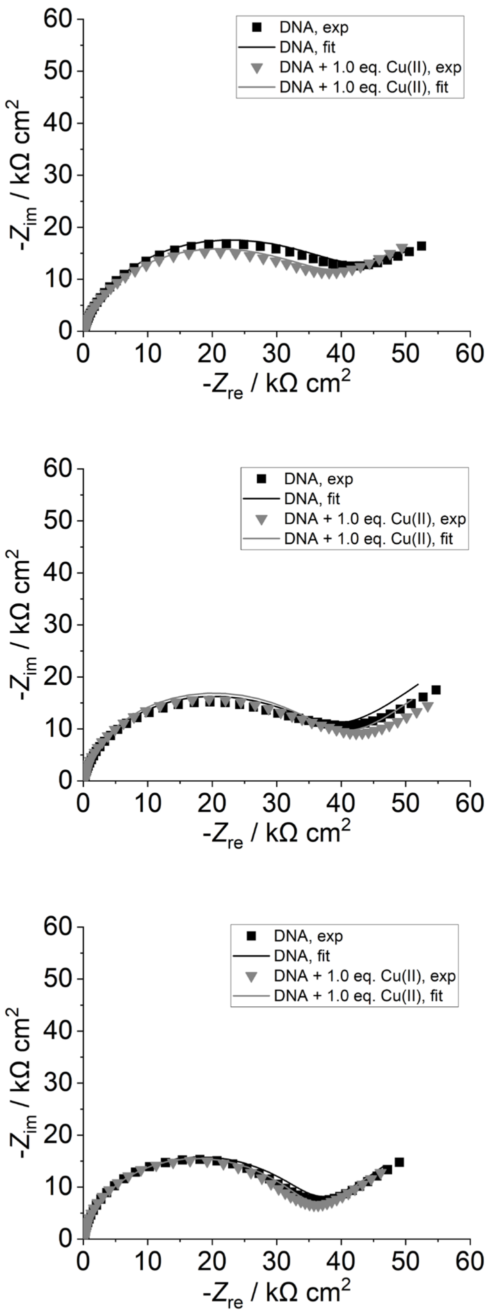

Figure S22: Representative Nyquist plot of the EIS data of a DNA film containing duplex **PH$_C$** (top), **PH$_M$** (center), and **PH$_F$** (bottom) immobilized on a gold electrode. The data were fitted using the modified Randles circuit shown in Figure 6. Experimental conditions: 2 mM $K_4[Fe(CN)_6]$, 2 mM $K_3[Fe(CN)_6]$, 5 mM MOPS (pH 6.8), 150 mM $NaClO_4$.

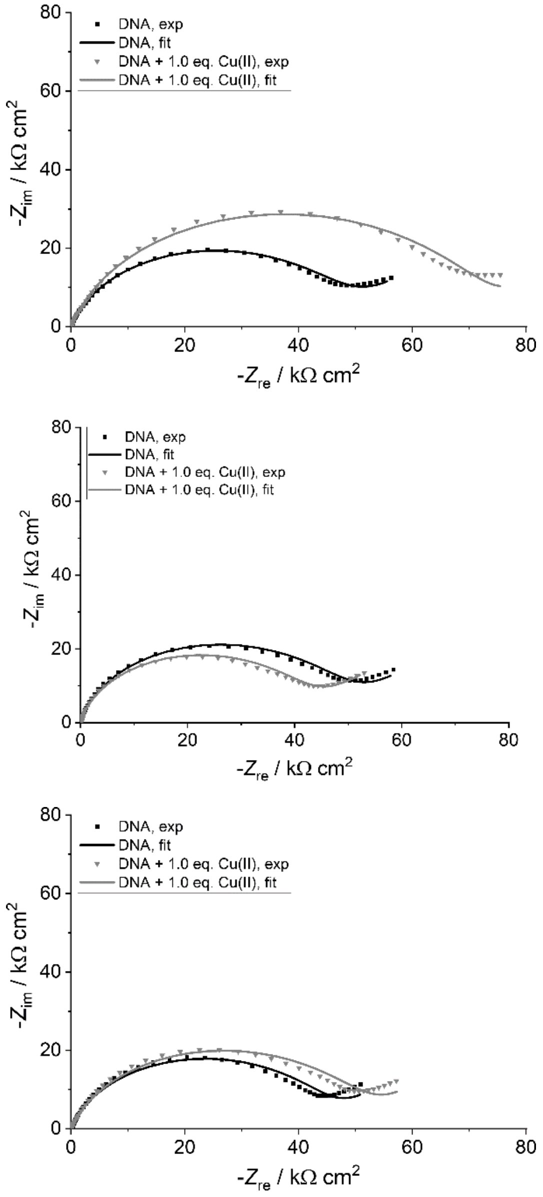

Figure S23: Representative Nyquist plot of the EIS data of a DNA film containing duplex **PP$_C$** (top), **PP$_M$** (center), and **PP$_F$** (bottom) immobilized on a gold electrode. The data were fitted using the modified Randles circuit shown in Figure 6. Experimental conditions: 2 mM $K_4[Fe(CN)_6]$, 2 mM $K_3[Fe(CN)_6]$, 5 mM MOPS (pH 6.8), 150 mM $NaClO_4$.

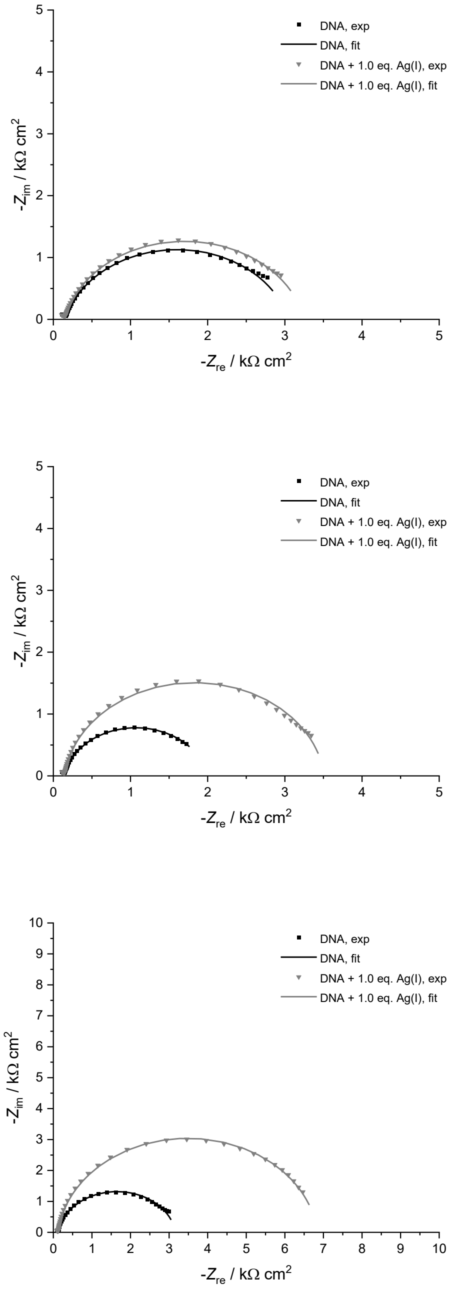

Figure S24: Representative Nyquist plot of the EIS data of a DNA film containing duplex **DD$_C$** (top), **DD$_M$** (center), and **DD$_F$** (bottom) immobilized on a gold electrode. The data were fitted using the modified Randles circuit shown in Figure 6. Experimental conditions: 2 mM $K_4[Fe(CN)_6]$, 2 mM $K_3[Fe(CN)_6]$, 5 mM MOPS (pH 6.8), 150 mM $NaClO_4$.

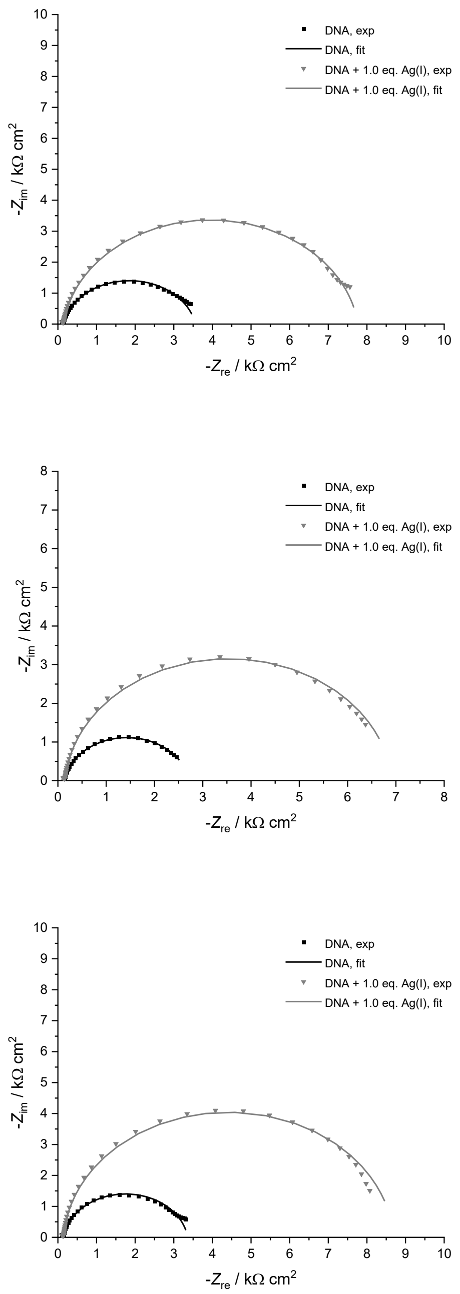

Figure S25: Representative Nyquist plot of the EIS data of a DNA film containing duplex **CD**$_C$ (top), **CD**$_M$ (center), and **CD**$_F$ (bottom) immobilized on a gold electrode. The data were fitted using the modified Randles circuit shown in Figure 6. Experimental conditions: 2 mM $K_4[Fe(CN)_6]$, 2 mM $K_3[Fe(CN)_6]$, 5 mM MOPS (pH 6.8), 150 mM $NaClO_4$.

Table S3: Individual equivalent circuit elements of the dsDNA film before and after Cu^{II} or Ag^{I} addition.[a]

		R_{CT} / kΩ cm²	*CPE* / μF	n_{CPE}	ΔR_{CT} / kΩ cm²
**HH$_C$**	0.0 eq. of Cu^{II}	38(2)	1.9(6)	0.79(3)	–1(4)
	1.0 eq. of Cu^{II}	37(3)	1.5(3)	0.81(2)	
**HK$_C$**	0.0 eq. of Cu^{II}	40(4)	1.2(3)	0.81(2)	–1(4)
	1.0 eq. of Cu^{II}	39(2)	0.8(4)	0.86(3)	
**HP$_C$**	0.0 eq. of Cu^{II}	96(5)	0.8(2)	0.84(2)	–29(6)
	1.0 eq. of Cu^{II}	66(4)	0.5(2)	0.86(4)	
**PH$_C$**	0.0 eq. of Cu^{II}	40(1)	0.8(3)	0.7(3)	–4(1)
	1.0 eq. of Cu^{II}	36(1)	0.7(2)	0.89(4)	
**PP$_C$**	0.0 eq. of Cu^{II}	68(2)	0.9(2)	0.86(4)	7(4)
	1.0 eq. of Cu^{II}	75(4)	0.9(3)	0.86(3)	
**DD$_C$**	0.0 eq. of Ag^{I}	3.4(7)	1.3(3)	0.84(2)	0(1)
	1.0 eq. of Ag^{I}	3.4(5)	1.0(3)	0.87(2)	
**CD$_C$**	0.0 eq. of Ag^{I}	4.0(4)	0.6(3)	0.90(3)	4.2(6)
	1.0 eq. of Ag^{I}	8.2(4)	0.5(2)	0.95(2)	
**HH$_M$**	0.0 eq. of Cu^{II}	54(3)	1.3(3)	0.82(1)	–2(4)
	1.0 eq. of Cu^{II}	51(2)	1.1(3)	0.82(1)	
**HK$_M$**	0.0 eq. of Cu^{II}	39(3)	1.0(3)	0.84(2)	1(4)
	1.0 eq. of Cu^{II}	39(3)	0.8(2)	0.85(2)	
**HP$_M$**	0.0 eq. of Cu^{II}	69(4)	0.8(4)	0.86(3)	–19(5)
	1.0 eq. of Cu^{II}	49(3)	0.9(5)	0.84(4)	
**PH$_M$**	0.0 eq. of Cu^{II}	46(2)	2(2)	0.91(2)	9(5)
	1.0 eq. of Cu^{II}	55(5)	0.8(4)	0.8(3)	
**PP$_M$**	0.0 eq. of Cu^{II}	50(2)	1.1(3)	0.88(3)	–8(4)
	1.0 eq. of Cu^{II}	42(2)	0.9(2)	0.88(3)	
**DD$_M$**	0.0 eq. of Ag^{I}	1.9(1)	0.8(3)	0.888(8)	1.4(2)
	1.0 eq. of Ag^{I}	3.3(2)	0.6(2)	0.931(5)	
**CD$_M$**	0.0 eq. of Ag^{I}	2.3(3)	0.52(4)	0.902(6)	3.6(9)
	1.0 eq. of Ag^{I}	5.9(9)	0.36(3)	0.950(3)	
**HH$_F$**	0.0 eq. of Cu^{II}	33(2)	1.3(4)	0.82(3)	–1(3)
	1.0 eq. of Cu^{II}	32(2)	1.0(3)	0.84(2)	
**HK$_F$**	0.0 eq. of Cu^{II}	28(3)	0.9(3)	0.85(3)	9(4)
	1.0 eq. of Cu^{II}	37(3)	0.7(2)	0.86(2)	
**HP$_F$**	0.0 eq. of Cu^{II}	44(3)	0.9(3)	0.85(4)	0(3)
	1.0 eq. of Cu^{II}	44(1)	0.7(2)	0.86(2)	

		R_{CT} / kΩ cm^2	*CPE* / μF	n_{CPE}	ΔR_{CT} / kΩ cm^2
**PH$_F$**	0.0 eq. of Cu^{II}	33(1)	0.8(3)	0.8(3)	–2(1)
	1.0 eq. of Cu^{II}	31(1)	0.6(2)	0.91(2)	
**PP$_F$**	0.0 eq. of Cu^{II}	43(2)	0.8(2)	0.90(1)	–1(3)
	1.0 eq. of Cu^{II}	42(2)	0.8(2)	0.89(2)	
**DD$_F$**	0.0 eq. of Ag^{I}	3.3(1)	0.7(1)	0.908(2)	3.5(4)
	1.0 eq. of Ag^{I}	6.8(4)	0.5(1)	0.943(7)	
**CD$_F$**	0.0 eq. of Ag^{I}	3.0(4)	0.50(4)	0.911(5)	5.3(5)
	1.0 eq. of Ag^{I}	8.3(5)	0.34(2)	0.958(5)	

[a] Values determined by using the equivalent circuit depicted in Figure 6. Solution resistance R_s is not included as it is negligibly small (<0.01 kΩ cm^2). The average of at least three individual measurements is given with its respective standard deviation.

Reference

[1] E.-K. Kim, C. Switzer, "Bis(6-carboxypurine)-Cu^{2+}: A Possibly Primitive Metal-Mediated Nucleobase Pair" *Org. Lett.* **2014**, *16*, 4059-4061.
